# Appraising the holistic value of Lenvatinib for radio-iodine refractory differentiated thyroid cancer: A multi-country study applying pragmatic MCDA

**DOI:** 10.1186/s12885-017-3258-9

**Published:** 2017-04-17

**Authors:** Monika Wagner, Hanane Khoury, Liga Bennetts, Patrizia Berto, Jenifer Ehreth, Xavier Badia, Mireille Goetghebeur

**Affiliations:** 1 LASER Analytica, Montreal, Quebec, Canada; 2 LASER Analytica, Milan, Italy; 3 LASER Analytica, Paris, France; 4 LASER Analytica and Omakase Consulting, Barcelona, Spain; 5 School of Public Health, University of Montreal, Montreal, Quebec, Canada

**Keywords:** Mcda, Appraisal, Healthcare decisionmaking, Lenvatinib

## Abstract

**Background:**

The objective of the study was to reveal through pragmatic MCDA (EVIDEM) the contribution of a broad range of criteria to the value of the orphan drug lenvatinib for radioiodine refractory differentiated thyroid cancer (RR-DTC) in country-specific contexts.

**Methods:**

The study was designed to enable comprehensive appraisal (12 quantitative, 7 qualitative criteria) in the current disease context (watchful waiting, sorafenib) of France, Italy and Spain. Data on the value of lenvatinib was collected from diverse stakeholders during country-specific panels and included: criteria weights (individual and social values); performance scores (judgments on evidence—collected through MCDA systematic review); qualitative impacts of contextual criteria; and verbal and written insights structured by criteria. The value contribution of each criterion was calculated and uncertainty explored.

**Results:**

*Comparative effectiveness*, *Quality of evidence* (Spain and Italy) and *Disease severity* (France) received the greatest weights. Four criteria contributed most to the value of lenvatinib, reflecting its superior *Comparative effectiveness* (16–22% of value), the severity of RR-DTC (16–22%), significant unmet needs (14–21%) and robust evidence (14–20%). Contributions varied by comparator, country and individuals, highlighting the importance of context and consultation. Results were reproducible at the group level. Impacts of contextual criteria varied across countries reflecting different health systems and cultural backgrounds. The MCDA process promoted sharing stakeholders’ knowledge on lenvatinib and insights on context.

**Conclusions:**

The value of lenvatinib was consistently positive across diverse therapeutic contexts. MCDA identified the aspects contributing most to value, revealed rich contextual insights, and helped participants express and explicitly tackle ethical trade-offs inherent to balanced appraisal and decisionmaking.

**Electronic supplementary material:**

The online version of this article (doi:10.1186/s12885-017-3258-9) contains supplementary material, which is available to authorized users.

## Background

Lenvatinib is a tyrosine kinase inhibitor (TKI), indicated for the treatment of patients with progressive, locally advanced or metastatic, differentiated thyroid carcinoma, refractory to radioactive iodine (RR-DTC). [1] The efficacy of lenvatinib was demonstrated in a large (*N* = 392) placebo-controlled, phase III clinical trial. Lenvatinib prolonged progression-free survival (PFS) by 14.7 months (18.3 vs 3.6 months; hazard ratio [HR] 0.21, 95% CI 0.14–0.31, *P* < .001) and significantly reduced the risk of death after adjustment for placebo patients’ cross-over (overall survival [OS] HR 0.53, 95% CI 0.34–0.82). [2, 3] The most frequent treatment-related adverse effects (AEs) were hypertension, diarrhea, fatigue or asthenia, decreased appetite, decreased weight and nausea, which were mostly managed with standard clinical interventions or dose modifications. [2].

Sorafenib, another TKI, is the only other medicine for RR-DTC approved in Europe. [4] In the absence of approved therapies, patients may be followed with watchful waiting or receive localized palliative treatments of metastases. [5–12] In clinical practice, a variety of chemotherapeutic agents as well as other TKIs are used off-label. [13].

Lenvatinib carries orphan drug designations for papillary and follicular thyroid cancers based on their rarity and debilitating and life threatening nature, and the significant benefit it provides. [14, 15].

Appraisal of new products for reimbursement, particularly orphan products, [16, 17] is challenging as it confronts decisionmakers with competing ethical demands: broadly responding to the imperative to alleviate and prevent suffering, exercising fairness by prioritizing those most in need, while ensuring efficient allocation of resources to maintain healthcare system sustainability. At the root of these appraisals is the identification and measurement of the holistic value of interventions, which requires a broader perspective than the current cost-effectiveness paradigm to capture all relevant aspects. [17].

Pragmatic multi-criteria decision analysis (MCDA) can enable holistic appraisals and helps reveal and tackle the ethical trade-offs between conflicting demands to facilitate accountable decisionmaking. [18–23] EVIDEM, an open-source MCDA framework, was designed to stimulate structured reflection and pragmatic collection of insights on the true value of interventions from all stakeholders, through a broad set of quantitative and qualitative criteria, each explicitly rooted in ethical aspects inherent to fair and accountable decisionmaking, [21, 24–26] Its flexible design allows to include scientific and colloquial evidence, and incorporate individual and social values and country-specific contexts.

The objectives of this study were to assess the contribution of a broad range of decision criteria to the value of lenvatinib for RR-DTC from the perspective of three country-specific panels representing a diversity of stakeholders using pragmatic MCDA.

## Methods

### Study design

**Fig. 1:**
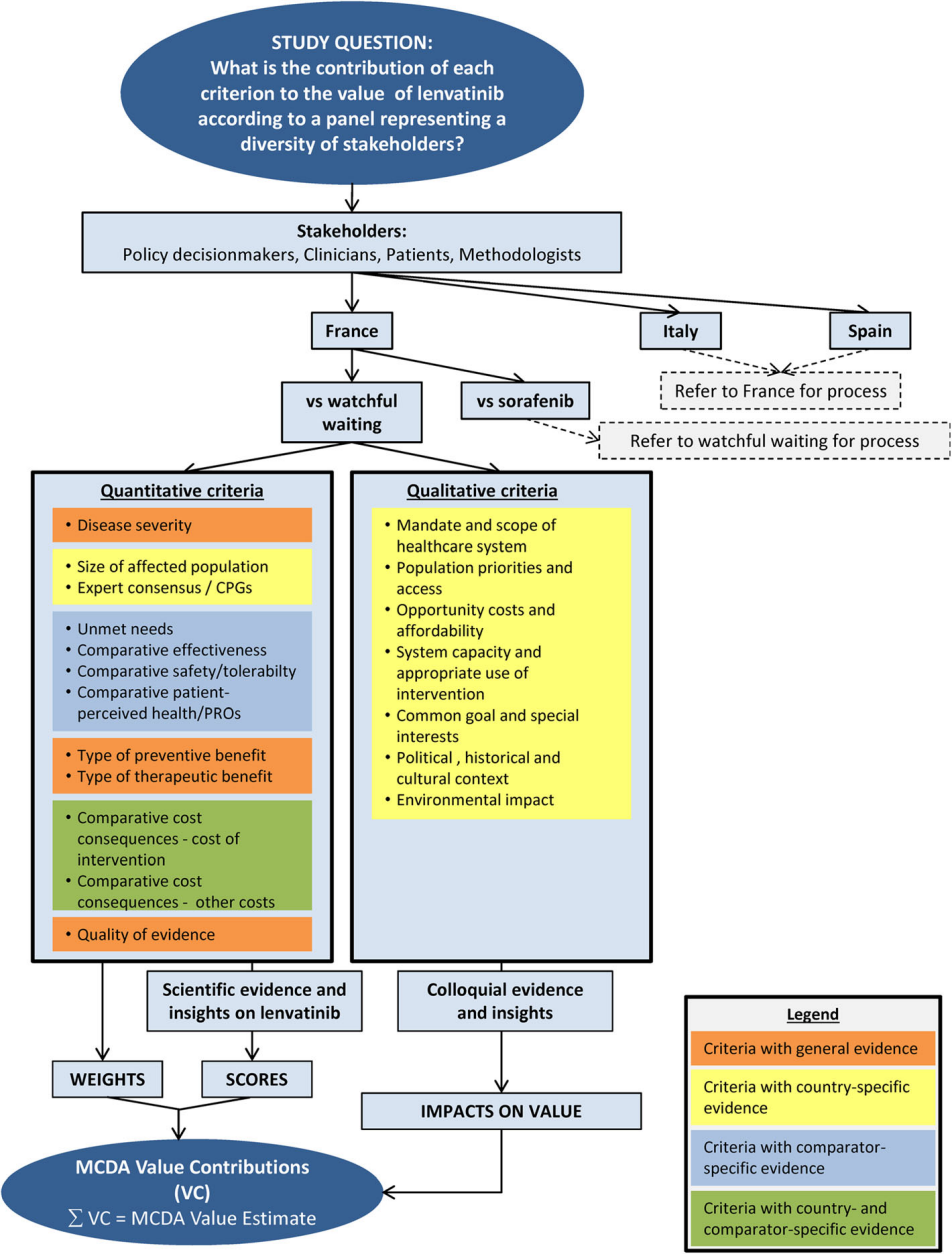
Study design

The study was designed based on analysis of the context in which lenvatinib will be appraised (Fig. 1). Comparators were interventions indicated for the systemic treatment of RR-DTC, which included sorafenib only. Since at the time of the assessment, reimbursement decisions for sorafenib had not yet been issued in target countries, watchful waiting was used as a second comparator. France, Italy and Spain were selected for country-specific assessments, as their HTAs involve multiple criteria. To collect insights from a broad range of perspectives and aim for a balanced appraisal, panels included a diversity of stakeholders. To explore the holistic value of lenvatinib, the EVIDEM framework (v2.4 available at time of study) was selected and all criteria were included (criteria definitions - Additional File 1).

### Evidence on lenvatinib: MCDA systematic literature review

MCDA Evidence Matrices were created using a systematic review protocol (included in the EVIDEM framework) for identification, analysis, synthesis and reporting of evidence following good HTA practice [27] adapted to provide necessary and sufficient evidence to appraise each criterion. Evidence was obtained from public and proprietary sources, including major biomedical literature databases (PubMed/Medline), Cochrane systematic reviews, clinical trial registries, cancer registries, conference websites (ISPOR, ASCO, ESMO) and proprietary data supplied by the manufacturer (Eisai). Additional sources were HTA agency reports, steering committees, pan-European and national/regional rare disease organizations, rare disease initiatives, thyroid cancer research networks and patient organizations (Additional File 2). The MCDA Evidence Matrix for lenvatinib was based on a total of 57 references. The matrices were tailored to each country’s context and translated into local languages (see English version of Italian context – Additional File 3).

### Panel design and conduct

Panels included policy decisionmakers, specialists, patient representatives, and methodologists with decisionmaking expertise, who were identified using predefined selection criteria (Additional File 4) and invited to participate in the study following local legal requirements. Three 1-day, country-specific panel sessions were conducted (each with 8 panelists) under the Chatham House Rule [28] to foster rich participation at each step. Panel sessions were chaired by experienced investigators who provided an introduction on MCDA, criteria of the framework and lenvatinib, and panelists were then guided to complete individually the MCDA Manual.

Panelists were first instructed to assign criteria weights independently of lenvatinib, to express their individual value system, from the perspective of coverage decisionmaking in their country. Weight elicitation was performed using a 5-point direct weighting scale for the primary analysis, [21] and hierarchical point allocation (HPA) for sensitivity analyses.

For assessment of lenvatinib, each criterion was explored sequentially using the country-specific MCDA Evidence Matrix. Panelists were encouraged to share their insights and knowledge with the group, then make their assessments individually and provide additional written comments. Each quantitative criterion was scored by panelists on constructed, cardinal scales, ranging from 0 to 5 for non-comparative and −5 to 5 for comparative criteria (Fig. 1). For contextual (qualitative) criteria, panelists provided their insights and indicated how their consideration impacted lenvatinib’s value. Additionally, oral and written feedback on the process and tools was collected.

### Exploration of uncertainty

Panelists were allowed to provide score ranges for each criterion to express their uncertainty in judging the evidence. To explore the impact of the weighting method, HPA, was used. [29] To assess reproducibility of weights, scores and value estimates, panelists repeated the exercise individually at least two weeks after the panel sessions (retest).

### Data analysis

Criteria weights, scores and impacts were analyzed as reported previously. [21] The value contribution (VC_x_) of each quantitative criterion was calculated as the product of its normalized weight (W_x_, ∑ W_x_ = 1) and standardized score (S_x_ = score/5). The overall MCDA value estimate (VE) is the sum of all criteria value contributions:

$$ \mathrm{VE}=\sum_{x=1}^{\mathrm{n}}{\mathrm{VC}}_x=\sum_{x=1}^n\left({\mathrm{W}}_x\times {\mathrm{S}}_x\right) $$

Value estimates obtained using different weighting methods were compared using t-tests, paired at the level of the individual panelist. Consistency of retest data was assessed by calculating intra-rater correlation coefficients (ICC 3,1) [30] for normalized weights, scores and value estimates as reported previously [21].

## Results

### Perspectives of stakeholders – Panelists’ weights

**Fig. 2:**
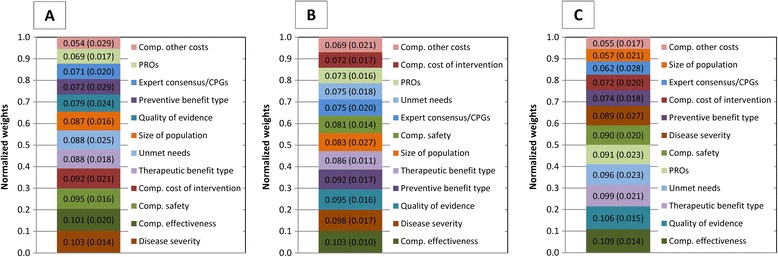
Mean (SD) normalised weights assigned to each quantitative criterion by the French (**a**), Italian (**b**), Spanish panels (**c**) using the 5-point direct weight elicitation technique

In each country, the top-3 highest ranking criteria accounted for 30 to 31% of the total weight: “Comparative effectiveness” (all 3 countries), “Disease severity” (France and Italy), “Quality of evidence” (Italy and Spain), “Comparative safety” (France) and “Type of therapeutic benefit” (Spain) (Fig. 2). The highest weighted criteria also tended to show the smallest standard deviations (SD) indicating a level of agreement on the most important criteria.

Although the study was not powered to measure variations across categories of stakeholders, exploratory analysis indicated that patient representatives tended to assign higher weights to “Comparative patient-perceived health/PROs” than other panelists, and lower to “Comparative cost” (Additional File 5).

### Performance scores based on evidence and panelists’ insights on lenvatinib

**Fig. 3:**
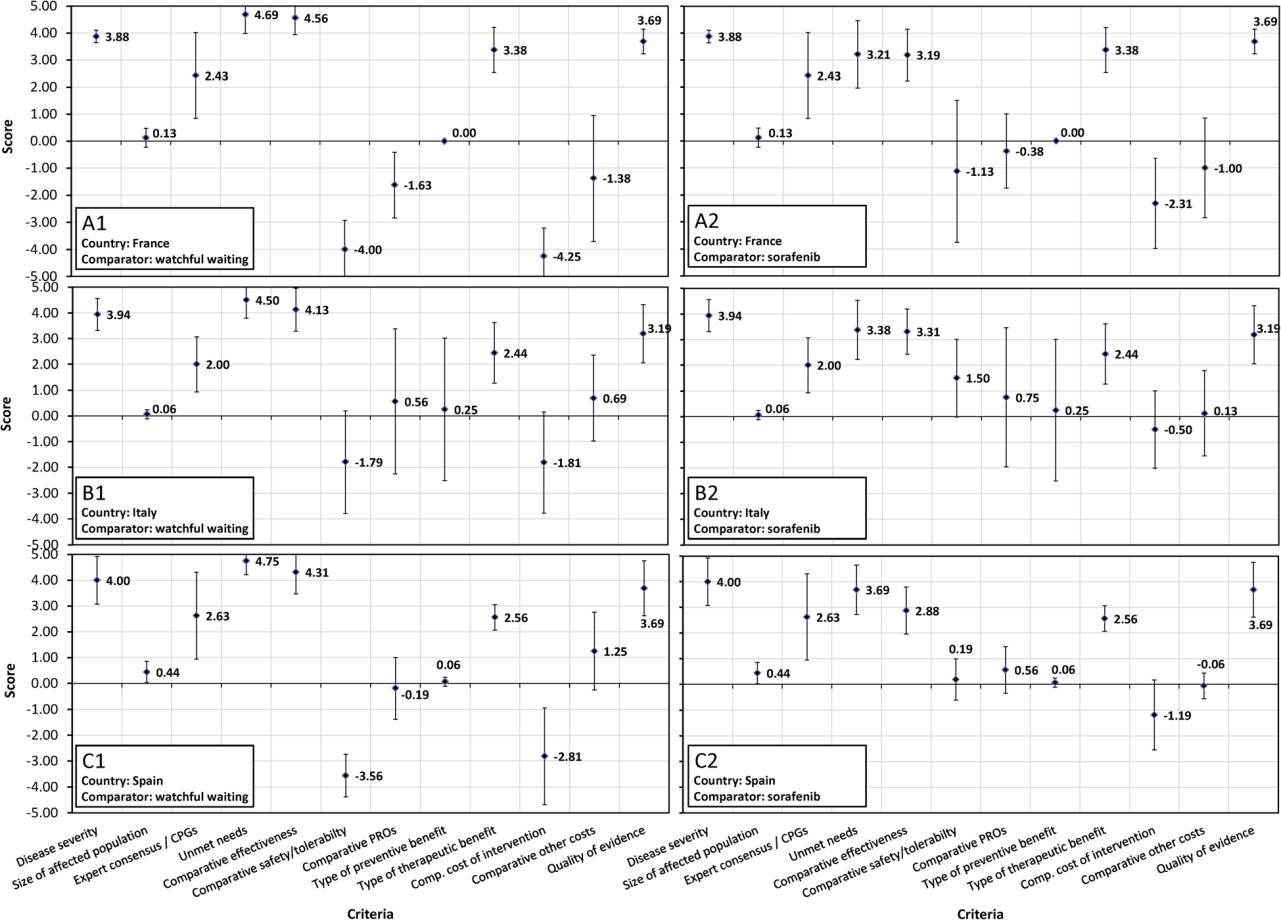
Mean (SD) scores for lenvatinib for RR-DTC assigned to each quantitative criterion by the French (**a**), Italian (**b**), and Spanish (**c**) panels versus watchful waiting (1) and sorafenib (2). A constructed, cardinal scoring scale was used, ranging from 0 to 5 for non-comparative and from −5 to 5 for comparative criteria

Below is a summary of the evidence on lenvatinib presented in the MCDA Evidence Matrix (Additional File 3), insights shared during group discussions that preceded individual scoring, individual written comments, and scores (Fig. 3) attributed by panelists based on all of the above, representing their judgements on the performance of lenvatinib. Detailed results are reported for Italy, with differences and similarities highlighted for France and Spain.

In Italy, severity of RR-DTC was scored 3.94 on a scale of 0 to 5, with good agreement among panelists (SD 0.62), reflecting panelists’ perception of its impact on mortality (approximately 19 months median OS of patients with progressive disease [2, 31, 32]) and morbidity (symptoms such as pain and airway obstruction leading to asphyxia [6, 33]). “Size of population” was scored close to 0, in line with the condition’s low estimated prevalence (3.8/100,000) and incidence (0.3/100,000 per year). [34–37] The strength of “Expert consensus/CPG recommendations” for lenvatinib [5–7, 9] was judged as moderate (mean score 2.00). Panelists noted that, when guidelines were published, lenvatinib had not yet received licensing approval; therefore, recommendations applied for the drug class and not specifically for lenvatinib, which may have affected their scoring. They commented that updated CPGs are expected to provide strong recommendations for lenvatinib. “Unmet needs” were judged as very high (mean score 4.50) when the comparator was watchful waiting. Panelists confirmed, in agreement with the evidence presented, [6] that traditional chemotherapy, although sometimes used, does not prolong PFS and OS in this population. “Unmet needs” were perceived as less pronounced but still high (mean score 3.38) when the availability of sorafenib (which improved PFS by 5.0 months in a placebo-controlled RCT [38]) was considered.

Lenvatinib’s “Comparative effectiveness” versus watchful waiting was considered high (mean score 4.13, scale −5 to +5), based on a 14.7-month improvement in PFS and reduced risk of death after adjustment for cross-over. [2, 3] One panelist commented that “the evidence on PFS is pretty strong and convincing” and another noted that “the OS data seems to be exceptional”. Lenvatinib’s “Comparative effectiveness” versus sorafenib was also judged to be significantly higher (mean score 3.31), based on indirect treatment comparison (ITC), as panelists noted that the PFS difference between these therapies (risk ratio: 0.38, 95% CI 0.24–0.58) was very relevant.

Lenvatinib’s “Comparative safety/tolerability” versus watchful waiting received a moderately negative score (mean − 1.79, scale −5 to +5), reflecting greater frequency of treatment-related serious adverse events (AEs) compared to placebo (30.3% vs 6.0%). [2] “Comparative safety/tolerability” versus sorafenib, assessed comparing data from two placebo-controlled RTCs, [2, 31] was judged in favor of lenvatinib (mean score 1.50). Panelists noted that the AE profiles of these agents differed, and that an important aspect was the reversibility of AEs. For both comparisons, panelists differed widely in their judgements (scores) of comparative safety (SDs 1.99 and 1.51).

Assessment of lenvatinib’s “Comparative PROs” also showed wide variations: although mean scores were slightly positive (vs watchful waiting: 0.56, vs sorafenib: 0.75), SDs were very large (2.82 and 2.71, respectively). Panelists had difficulty assessing this criterion using the evidence available, which included PRO data from the sorafenib RCT (small negative effect on QoL compared to placebo [38]) and utilities from a vignette study of the UK general population (modestly positive utility impact of response to therapy, negative impacts of specific TKI toxicities [39]). The relevance of the utility study was discussed: some noted that the QoL impact of toxicities may differ between the general population and patients with RR-DTC; others pointed out that beyond global QoL, one should focus on specific outcomes relevant to patients. The discussion highlighted the need for QoL/PRO data for lenvatinib.

For “Quality of evidence”, panelists assigned a mean score of 3.19 based on an analysis of quality of the lenvatinib clinical program performed by the investigators. Comments included that the program featured a robust phase III study with a well-defined patient population, that evidence on PFS was strong and convincing, and that the ITC data seemed robust. However, there was concern about the lack of patient PRO data for lenvatinib and the effect of crossover on the validity of the OS data. One panelist commented that to receive the maximum score of 5 “there must be outcomes data reported by the patient and something definitive in terms of OS.”

With respect to the type of benefit that lenvatinib provides, panelists’ scores reflected that extending life but not curing the disease, it represents a treatment that provides a moderate type of therapeutic benefit (mean 2.44).

Lenvatinib’s “Comparative cost” was assessed based on manufacturer’s budget impact (BI) models. For Italy, these indicated an incremental cost versus watchful waiting of €19–24 million per year over five years, reflecting that lenvatinib would displace lower-cost off-label therapies (e.g., doxorubicin) and allow treating more patients. Taking the availability of sorafenib into account, the incremental impact was estimated at €13–17 million per year. Panelists commented that the low incidence of RR-DTC limited its BI, which they judged, on average, as moderate to low, with mean scores of −1.81 versus watchful waiting and −0.50 versus sorafenib, with large variations (SD 1.96 and 1.51, respectively). Lenvatinib’s “Comparative other costs”, which included potential savings due to fewer hospitalizations and physician visits, was judged, on average, as positive but small.

There was general agreement across the three countries on assessment of “Disease severity”, “Size of population”, “Unmet needs”, “Comparative effectiveness”, “Type of preventive”, “Type of therapeutic benefit” and “Quality of evidence”, with less than 1 unit difference in mean scores across the three panels (Fig. 3). However, both French and Spanish panels judged “Comparative safety/tolerability” versus watchful waiting more negatively than the Italian panel (mean scores −4.00 and −3.56, respectively) and tended to judge safety versus sorafenib more negatively as well (−1.13 and 0.19). French panelists assigned a negative score to “Comparative PROs” versus watchful waiting (−1.63), commenting that the UK general population utility study would not be accepted in France. They also viewed lenvatinib’s BI less favorably (vs watchful waiting: −4.25; vs sorafenib: −2.31) and expressed uncertainty about the evidence for “Comparative other costs” due to the high number of variables.

### Value contributions and estimates

**Fig. 4:**
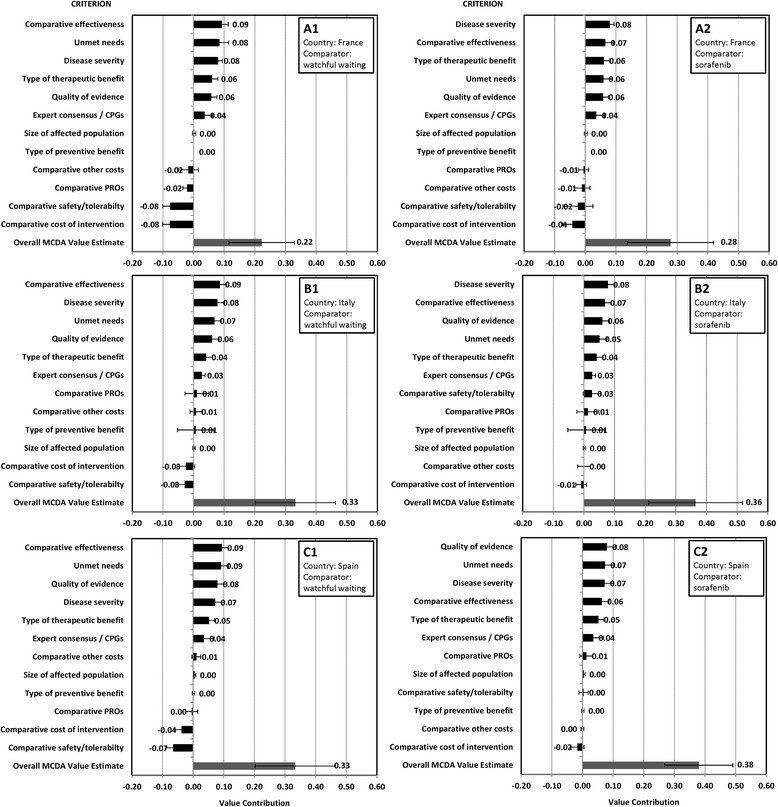
Mean value contributions* of each quantitative criterion and overall MCDA value estimates† for lenvatinib for RR-DTC from the French (**a**), Italian (**b**), and Spanish (**c**) panels versus watchful waiting (1) and sorafenib (2). * Value contribution = Normalized weight ×standardized score; †Overall Value Estimate = ∑ Value contribution of all 12 criteria. Error bars show standard deviations across 8 panelists in each country-specific panel

Figure 4 shows the criteria contributions to the value of lenvatinib by country and comparator. All three country panels judged lenvatinib as adding value (i.e., positive value estimate) compared to watchful waiting or sorafenib.

With watchful waiting as comparator, over 50% of positive value contributions were made by three criteria: “Comparative effectiveness” (21–22%) and “Unmet needs” (18–21%) in all three countries, together with “Disease severity” (19–20%) in France and Italy and “Quality of evidence” (18%) in Spain. “Comparative cost” and “Comparative safety/tolerability” contributed the most to reducing the value of lenvatinib, particularly in France. The MCDA value estimate for lenvatinib was 0.33 (SD 0.13) in both Italy and Spain and 0.22 (SD 0.11) in France.

With sorafenib as comparator, the top-3 value contributors were “Disease severity” (18–22%, all countries), “Comparative effectiveness” (18%, France and Italy), “Quality of evidence” (16–20%, Italy and Spain), “Type of therapeutic benefit” (17%, France) and “Unmet needs” (18%, Spain). “Comparative cost” negatively contributed to value in all three countries. The MCDA value estimate was 0.36 (SD 0.15) in Italy, 0.38 (SD 0.11) in Spain and 0.28 (SD 0.14) in France.

### Qualitative assessment of lenvatinib – Impacts of contextual criteria

**Fig. 5:**
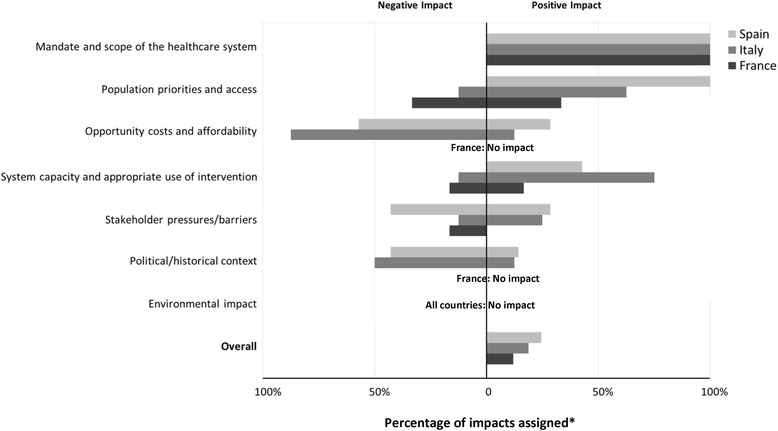
Impacts of contextual criteria on the appraisal of lenvatinib assigned by panelists in France, Italy and Spain, as percentage of impacts assigned. *Percentage of impacts (positive or negative) of all impacts assigned for a given criterion; Overall impact across criteria = (∑ Positive impacts - ∑ Negative impacts) / ∑ all impacts assigned

Italian panelists agreed that lenvatinib, being a treatment for cancer, was aligned with the “mandate and scope of their healthcare system”, with a positive impact on its value (Fig. 5). The “Population priorities and access” criterion (based on the fairness principle) included the consideration that lenvatinib targets a rare disease. The majority of Italian panelists thought that this would have a positive impact on value. Comments included that patients with rare disease should have a right to receive appropriate therapies when available. However, some panelists pointed out that, apart from recent changes in the Italian AIFA process, rare diseases do not really have special status in their healthcare system, while another commented that other factors need to be prioritized. Consideration of “Opportunity costs and affordability” had a negative impact on lenvatinib’s value, as panelists noted that its adoption would require disinvestments in other healthcare areas, which are more and more of concern. Another panelist commented that potential alternatives are less efficacious and savings, stemming from reduced hospitalization and productivity losses, need to be considered.

With respect to “System capacity and appropriate use”, most Italian panelists agreed that their health system was prepared to ensure appropriate use of lenvatinib. “Stakeholder pressures and barriers” were thought by the majority to have no impact on the value of lenvatinib. Half of panelists considered that “Political, historical & cultural context,” would have a negative impact, noting that in Italy the drug may be subject to a risk sharing agreement, such as payment by results. Overall, consideration of the contextual criteria had a positive impact on the value of lenvatinib in Italy.

Spanish panelists agreed that consideration of “Population priorities and access” had a positive impact on lenvatinib’s value (Fig. 5); however, they noted that currently there is no prioritization of orphan drugs in Spain. They were generally confident that their healthcare system could appropriately implement the use of lenvatinib. Contextual considerations had an overall positive impact on lenvatinib’s appraisal in Spain.

French panelists expressed a diversity of views regarding prioritization of rare diseases, with the majority indicating no or negative impact (Fig. 5). Most of the other context-specific criteria, except “Mandate and scope of the healthcare system”, were thought to have no or mixed impact on lenvatinib’s appraisal in France.

### Exploration of uncertainty

Use of the HPA weighting method increased the mean (group) value estimates by less than 5% in Italy (absolute increases <0.02) and, depending on comparator, by 15 to 24% in France and Spain (absolute increases 0.04–0.08). These differences were not statistically significant (paired *t*-tests *P* > .05).

Criteria most frequently assigned ranges of scores reflecting uncertainty of judgement, included “Type of therapeutic benefit”, “Comparative effectiveness”, “Expert consensus/CPGs”, “Unmet needs” versus sorafenib, and “Comparative other costs”.

Value estimates showed good reproducibility on the panel level: for 7 out of 12 test-retest pairs, the difference was 0.02 or less (Table 1); only two differed by 0.09 to 0.10. ICCs (3,1) ranged from 0.437 to 0.913 across the 12 test-retest pairs, indicating moderate to good reproducibility on an individual panelist level. HPA generally led to better reproducible value estimates than the 5-point weighting scale. Reproducibility of scores was generally better than that of weights.

**Table 1:** Comparison of value estimates obtained in tests and re-tests^a^, by weighting technique, comparator and country

Comparator	Weighting technique	France		Italy		Spain	
		Mean test (SD)	Mean re-test (SD)	Mean test (SD)	Mean re-test (SD)	Mean test (SD)	Mean re-test (SD)
vs watchful waiting	5-point weighting scale	0.22 (0.14)	0.32 (0.13)	0.33 (0.14)	0.31 (0.10)	0.30 (0.11)	0.29 (0.07)
		ICC (3,1) = 0.732		ICC (3,1) = 0.511		ICC (3,1) = 0.628	
	Hierarchical point allocation	0.24 (0.13)	0.33 (0.17)	0.34 (0.15)	0.36 (0.20)	0.38 (0.20)	0.31 (0.15)
		ICC (3,1) = 0.913		ICC (3,1) = 0.668		ICC (3,1) = 0.877	
vs sorafenib	5-point weighting scale	0.31 (0.14)	0.29 (0.11)	0.35 (0.16)	0.34 (0.12)	0.36 (0.11)	0.31 (0.09)
		ICC (3,1) = 0.832		ICC (3,1) = 0.437		ICC (3,1) = 0.724	
	Hierarchical point allocation	0.32 (0.16)	0.30 (0.18)	0.37 (0.15)	0.36 (0.20)	0.41 (0.18)	0.33 (0.15)
		ICC (3,1) = 0.772		ICC (3,1) = 0.649		ICC (3,1) = 0.865	

*ICC* intra-rater correlation coefficient, *SD* standard deviation

^a^Test-retest data were available for 5 panelists from France, 7 from Italy and 7 from Spain

### Panelists’ feedback on process

Panelists reported that the process contributed to their understanding of the intervention and its context and was helpful for sharing their knowledge and understanding others’ perspectives. They noted that the method deepened the discussion and allowed explicit and comprehensive consideration of all relevant elements, beyond efficacy, safety and cost. The process yielded quantitative results that had high face validity. Several panelists commented that the design of the framework, with each criterion rooted in an ethical aspect, was useful in identifying the ethical trade-offs they had to make.

## Discussion

Applying comprehensive and pragmatic MCDA, systematic collection of quantitative and qualitative inputs, including group discussions and individual comments, allowed a deep exploration of the diverse aspects impacting the value of lenvatinib: the therapeutic context of RR-DTC, the evidence available, the values at stake, and the specific context of appraisals.

Across countries and comparators, four criteria contributed most to the value of lenvatinib: “Comparative effectiveness”, “Disease severity”, “Unmet needs” and “Quality of evidence”. “Comparative cost of intervention” and “Comparative safety” (vs watchful waiting) contributed negatively to value. The overall value of lenvatinib was positive in all analyses, with variability across individuals and countries (e.g., lower value estimate in France), pointing to the impact of individual perspectives and different cultural backgrounds. The EVIDEM value scale is rooted in the triple aim of healthcare, [40] i.e., doing what is best for patients, populations and healthcare systems, which have been integrated into the criteria and the design of the framework [41]. Therefore, as previously defined: [26] “The maximum value of 1 represents a hypothetical (ideal) intervention that prevents and cures severe endemic diseases with significant unmet needs and that, compared to existing approaches, has demonstrated large improvements in efficacy, safety and PROs as well as positive economic consequences.” Thus, lenvatinib’s value estimates reflect an intervention for a severe rare disease with significant unmet needs that has demonstrated large improvements in efficacy, limited value from safety and PROs, and some additional costs. The lower value estimates in France stem from a less favorable assessment of “Comparative cost” and “Comparative safety/tolerability”, combined with higher weights assigned to these criteria. Value estimates derived using different weighting methods did not differ significantly, confirming the robustness of the assessment. Value estimates on a panel level were generally similar between test and retest, supporting reproducibility of the appraisals. Panelists’ feedback underscored the comprehensiveness of the approach, the high face validity of the results and the usefulness of the reflection it triggered.

Appraisals derived from the EVIDEM methodology have been completed for a number of interventions, including drugs, devices and diagnostic tests, with value estimates ranging between 0.22 and 0.72 [20, 21, 25, 42–44]. Because this study used negative scales for all comparative criteria (EVIDEM v2.4), its value estimates are not comparable with those previously obtained. Indeed, the usefulness of this exercise lies more in identifying the contribution of each criterion to value and collecting contextual insights in a structured manner rather than the actual value estimate, which lacks a standardized frame of reference. Value estimates become useful when the MCDA framework is applied systematically by a given institution, such as in Lombardy, [45] where it provides a consistent, accountable and reasonable decisionmaking process allowing for prioritization of interventions that have the highest value in contributing to the triple aim.

Weighting revealed that criteria, often not explicitly considered in appraisal processes such as disease severity, are important, confirming results from large surveys among healthcare decisionmakers and stakeholders. [43, 46] Consideration of disease severity is rooted in distributive justice and fairness [26] and rank high in each country. It also revealed the predominance of the “imperative to help, an aspect of deontology including beneficence and non-maleficence” embedded in criteria “Effectiveness”, “Safety” and “Type of benefit”, which ranked among the highest weights. Panelists indicated the usefulness of being aware of the ethical underpinning of criteria to make a balance and meaningful appraisal in line with their values and the values they expect from their country institutions. Panelists’ individual value systems were reflected in the variation of weights, highlighting the critical impact of appraisal committee composition. Patient involvement in decisionmaking over a product’s life cycle is a much debated and researched topic, [47–54] particularly in the field of orphan diseases [49, 50, 53, 54]. Reflective MCDA approaches are well suited to capture the diversity of perspectives, enhance participation and communication, and improve understanding of the ethical trade-offs and dilemmas inherent in decisionmaking and resource allocation.

The scoring exercise showed a broad consensus in judgments on the severity of RR-DTC and the limitations of current treatments. There was also general agreement that lenvatinib provides major improvements in efficacy over watchful waiting as well as over sorafenib. Also, although their assessments varied in degree, all panelists agreed that the toxicity profile of lenvatinib was a limitation (negative contribution to value). In the SELECT trial, grade 3 or higher toxicities were seen in 75% of patients, resulting in dose reductions, dose interruptions and discontinuations in 67%, 82%, and 14% of patients, respectively. The most frequent grade 3 or higher treatment-related AEs were hypertension (42%), proteinuria (10%), fatigue (9%), diarrhea (8%), arterial and venous thromboembolic effects (2.7% and 3.8%, respectively), acute renal failure (1.9%), and hepatic failure (0.4%). [2] To mitigate these risks, regular monitoring of blood pressure, urine protein, clinical symptoms or signs of cardiac decompensation, liver function, electrolyte abnormalities, and TSH levels is required (see Additional File 3 – MCDA Evidence Matrix). [1] Also, the panelists were informed that a global study will be conducted to evaluate the efficacy and safety of a lower (< 24 mg once daily) lenvatinib starting dose (see Additional File 3 – MCDA Evidence Matrix). According to current NCCN guidelines, patients with progressive and/or symptomatic disease may be considered for lenvatinib therapy. [55] Broadly in line with this guideline, after reviewing the safety of lenvatinib a recent expert review recommended starting lenvatinib therapy in patients with symptomatic disease and those with rapid radiological or clinical disease progression. [56] In patients who are not yet symptomatic, lenvatinib’s potential to markedly reduce disease progression should be weighed against its potential toxicity [56].

Where evidence was lacking, limited or ambiguous, such as for PRO outcomes, clinical guidelines (which at the time of the study had not been updated), and some economic outcomes, performance scores typically differed widely (or score ranges were assigned), reflecting uncertainty in judging the evidence. Discussions with stakeholders allowed identifying which evidence is acceptable and most useful for a specific country, as related to cultural values. For example, French panelists noted that PFS data were very strong and relevant, while PROs derived from the general population are irrelevant in the French context.

Contextual criteria were considered qualitatively in this study in terms of type of impact on overall value; however, some of these criteria could be quantitatively operationalized in specific contexts [26]. Consideration of contextual criteria impacted lenvatinib’s appraisal positively in all three countries; however, country-specific differences were noticed: Consideration of “Population priorities and access”, mainly focusing on the rare disease status of RR-DTC, had a predominantly positive impact in Italy and Spain, but mixed impacts in France. Unlike French panelists, most Italian and Spanish panelists were confident in the ability of their healthcare systems to use lenvatinib appropriately, which had a positive impact on its value.

The study had some limitations. The 8-member panels were too small to be regarded as representative of their country. Clearly, individuals vary in their assessments, which may be influenced by personal and professional factors, such as experience, role in society and education. This study was not designed to investigate the impact of these factors on assessments; however, it included a diversity of stakeholders in an attempt to capture a broad variety of perspectives (see Additional File 4). On the other hand, the small panel size facilitated group discussions and sharing of comments, which allowed a more in-depth analysis of the different aspects involved. Another potential limitation is that misinterpretation of some evidence or a scoring scale may have occurred, in some cases resulting in scores that did not represent the true view of the panelist. In addition, for certain aspects of the appraisal, lack of relevant or up-to-date evidence (e.g., guidelines) may have impacted the assessments.

## Conclusion

The value of lenvatinib was assessed consistently as overall positive across diverse therapeutic landscapes, although limitations due to toxicity and costs were clearly noted. The process identified which criteria were most important to stakeholders and contributed most to value in each local context. The structuring and clarifying power of MCDA enabled collecting country- and comparator-specific data, increased exchange, and facilitated identifying the trade-offs that need to be made. Such rich content at the criterion level is required to understand where value lies to enhance communication between stakeholders and fully support reimbursement applications and decisionmaking in local contexts. Future research is needed to explore the value of lenvatinib in other settings and further develop MCDA processes across the decision continuum.

## Additional files

Additional file 1: Criteria definitions. (DOCX 37 kb)

Additional file 2: Targeted systematic literature review methodology. (DOCX 41 kb)

Additional file 3: MCDA evidence matrix for lenvatinib in the Italian context. (DOCX 49 kb)

Additional file 4: Recruitment criteria for panelists. (DOCX 31 kb)

Additional file 5: Exploratory analysis of weights by category of panelists. (DOCX 33 kb)

## Abbreviations

AE: adverse effect
BI: budget impact
HTA: health technology assessment
MCDA: multicriteria decision analysis
OS: overall survival
PFS: progression-free survival
PRO: patient-reported outcome
RCT: randomized-controlled trial
RR-DTC: radioactive iodine-refractory differentiated thyroid cancer
SD: standard deviation
