# Supplementary material for: Appraising the holistic value of Lenvatinib for radio-iodine refractory differentiated thyroid cancer: A multi-country study applying pragmatic MCDA
Source: BMC Cancer. 2017 Apr 17;17:272. doi: 10.1186/s12885-017-3258-9 (PMC5393009; doi:10.1186/s12885-017-3258-9)
Supplement: Supplementary file 1 — Criteria definitions. (DOCX 37 kb) [file 12885_2017_3258_MOESM1_ESM.docx]

# Additional File 1: Criteria Definitions

| Criteria | Definitions |
| --- | --- |
| Disease impact | |
| Disease severity | Severity of the health condition of patients treated with the proposed intervention (or severity of the health condition that is to be prevented) with respect to mortality, disability, function, impact on quality of life, clinical course (i.e., acuteness, clinical stages). |
| Size of affected population | Number of people affected by the condition (treated or prevented by the proposed intervention) among a specified population at a specified time; can be expressed as annual number of new cases (annual incidence) and/or proportion of the population affected at a certain point of time (prevalence). |
| **Context of intervention** | |
| Expert consensus/Clinical practice guideline recommendations (CPGs) | Concurrence of the proposed intervention (or similar alternatives) with the current consensus of experts on what constitutes state-of-the-art practices in the management of the targeted health condition; guidelines are usually developed via an explicit process and are intended to improve clinical practice. |
| unmet needs | Shortcomings of comparative interventions in their ability to prevent, cure, or ameliorate the condition targeted; also includes shortcomings with respect to safety, patient reported outcomes and convenience. |
| comparative outcomes of intervention (extent of benefit) | |
| Comparative effectiveness | Capacity of the proposed intervention to produce a desired (beneficial) change in signs, symptoms or course of the targeted condition above and beyond beneficial changes produced by alternative interventions. Includes efficacy and effectiveness data, as available. |
| Comparative safety / tolerability | Capacity of the proposed intervention to produce a reduction in intervention-related harmful or undesired health effects compared to alternative interventions. |
| Comparative patient-perceived health /patient- reported outcomes (PROs) | Capacity of the proposed intervention to produce beneficial changes in patient-reported outcomes (PROs) (e.g., quality of life) above and beyond beneficial changes produced by alternative interventions; also includes improvement in convenience to patients. |
| **Type of health benefit of intervention** | |
| Type of preventive benefit | Disease risk reduction provided by the proposed intervention at the population-level (e.g., prevention, reduction in disease transmission, reduction in the prevalence of risk factors). Public health perspective. |
| Type of therapeutic benefit | Nature of the clinical benefit provided by the proposed intervention at the patient-level (e.g., symptom relief, prolonging life, cure). |
| Economic consequences of intervention | |
| Comparative cost consequences ­­­– Cost of intervention | Net cost of covering the intervention (excluding other spending). This represents the differential between expected expenditures for the proposed intervention and potential cost savings that may result from replacement of other intervention(s) currently covered by the health plan. Limited to cost of intervention (e.g., acquisition cost, implementation and maintenance cost). |
| Comparative cost consequences ­­­– Other costs | Impact of providing coverage for the proposed intervention on other medical and non-medical costs (excluding intervention cost), such as hospitalization, specialist consultations, adverse event costs, long-term care, disability costs, lost productivity, caregiver time, etc. |
| Quality / uncertainty of evidence | |
| Quality of evidence | Extent to which evidence on the proposed intervention is relevant to the decisionmaking body (in terms of population, disease stage, comparator interventions, outcomes etc.) and valid with respect to scientific standards (i.e., study design etc.) and conclusions (agreement of results between studies). This includes consideration of uncertainty (e.g., conflicting results across studies, limited number of studies & patients). Consistent and complete reporting of evidence is a pre-requisite to assess validity. |
| Context and implementation* | |
| Mandate and scope of healthcare system | Alignment of the intervention with the mandate/scope of the healthcare system. The goal of healthcare is to maintain normal functioning. The mission and scope of health plans/systems derive from this principle. |
| Population priorities & access (principle of fairness) | Alignment of the intervention with current priorities of health system/plan. Priorities for specific groups of patients are defined by societies/decisionmakers and reflect their moral values. Such considerations are aligned with the principle of fairness, which considers treating like cases alike and different cases differently and often gives priority to those who are worst-off (theory of justice). |
| Opportunity costs & affordability | Consideration of the medical resources that may be forgone (opportunity costs) if the intervention is implemented and whether the healthcare system can afford implementing the intervention. Both opportunity cost and affordability considerations require a financial/budgeting exercise. Opportunity cost and affordability can be considered at the system/institution level and at the patient level. |
| System capacity & appropriate use of intervention | The capacity of a healthcare system to implement the intervention and to ensure its appropriate use depends on its infrastructure, organization, skills, legislation, barriers and risks of inappropriate use. Such considerations include mapping current systems and estimating whether the use of the intervention under scrutiny requires additional capacities (note: if available, economic data on these aspects could be included under the economic criterion of the MCDA model). |
| Common goal and specific interests | Pressures or barriers from groups of stakeholders or individuals are often part of the context surrounding healthcare interventions. Being aware of pressures and interests at stake and how they may affect decisionmaking helps ensure that decisions are fair-minded. |
| Political, historical and cultural context | The political, historical and cultural context may influence the value of an intervention with respect to specific political situations and overall priorities (e.g., priority for innovation) as well as habits, traditions and precedence. |
| Environmental impact | The extent to which the production, use or implementation of the intervention causes environmental damage. |

*These criteria are considered qualitatively using the MCDA contextualization tool.
