# Supplementary material for: Appraising the holistic value of Lenvatinib for radio-iodine refractory differentiated thyroid cancer: A multi-country study applying pragmatic MCDA
Source: BMC Cancer. 2017 Apr 17;17:272. doi: 10.1186/s12885-017-3258-9 (PMC5393009; doi:10.1186/s12885-017-3258-9)
Supplement: Supplementary file 2 — Targeted systematic literature review methodology. (DOCX 41 kb) [file 12885_2017_3258_MOESM2_ESM.docx]

# Additional File 2: Targeted Systematic Literature Review Methodology

## Search algorithms

**Sources and database**

Sources of information included:

- biomedical literature databases
- organization and individual country websites
- bibliographies of full text articles retrieved
- Non-public domain data provided by the developer (EISAI).

**Databases**

- PubMed / Medline
- NHS Centers for Review and Dissemination (CRD-DARE)
- Cochrane
- Trial registries

Table: Search algorithms

| **Searches** | **Searched terms:** | **No. of records retrieved** |
| --- | --- | --- |
| **Topic: impact on mortality, morbidity and QoL** | |  |
|  | (differentiated thyroid cancer OR radioiodine-refractory thyroid cancer OR Hurthle cell OR follicular thyroid cancer OR papillary thyroid cancer) AND (symptoms OR etiology OR aetiology OR presentation OR manifes* OR co-morbid* OR comorbid* OR “risk factor” OR “risk factors” OR progression)  *Limits: Review; published in the last 10 years; English;*  *French; Core clinical journals* | ***84*** |
|  | (differentiated thyroid cancer OR Hurthle cell OR follicular thyroid cancer OR radioiodine-refractory thyroid cancer OR papillary thyroid cancer) AND (death OR mortality)  *Limits: Published in the last 10 years; English; French; Review; Core clinical journals* | ***320*** |
|  | (differentiated thyroid cancer OR Hurthle cell OR follicular thyroid cancer OR radioiodine-refractory thyroid cancer OR papillary thyroid cancer) AND (quality of life OR QoL OR utility OR utilities OR burden OR HRQOL OR health-related quality of life OR EQ-5D OR EuroQuol-5D OR SF-36 OR FACT-Leu OR Functional Assessment of Cancer Therapy OR EORTC-QLQ-C30)  *Limits: published in the last 10 years; English; French;*  *Core clinical journals* | ***80*** |
|  | **Total articles selected** | ***42*** |
| **Topic: epidemiology** |  |  |
|  | (differentiated thyroid cancer OR Hurthle cell OR follicular thyroid cancer OR radioiodine-refractory thyroid cancer OR papillary thyroid cancer) AND (incidence OR prevalence OR epidemiolog*) AND (Spain OR Spanish OR France OR French OR Italy OR Italian) NOT (testosterone OR reactive oxygen species OR nanoparticle OR kinase)  *Limits: Published in the last 5 years* | ***219*** |
|  | **Total articles selected** | ***14*** |
| **Topic: clinical guidelines** |  |  |
|  | (differentiated thyroid cancer OR Hurthle cell OR follicular thyroid cancer OR radioiodine-refractory thyroid cancer OR papillary thyroid cancer OR thyroid cancer) OR (thyroid AND neoplasm)  *Limits: Published from 2012/03/01; Guideline; Practice Guideline* | ***13*** |
|  | (differentiated thyroid cancer OR Hurthle cell OR follicular thyroid cancer OR radioiodine-refractory thyroid cancer OR papillary thyroid cancer OR thyroid cancer) AND (guideline OR guidelines OR statement  OR consensus OR recommendation) NOT (primer OR primers)  *Limits: Publication date from 2012/03/01* | ***442*** |
|  | **Total articles selected** | ***19*** |
|  |  |  |
| **Topic: current treatment and unmet needs** | | |
|  | (differentiated thyroid cancer OR Hurthle cell OR follicular thyroid cancer OR radioiodine-refractory thyroid cancer OR papillary thyroid cancer) AND (“treatment pattern” OR “treatment patterns” OR  management OR "treatment practice" OR "therapeutic approach") NOT (rat OR rodent OR mouse OR mice OR murine)  *Limits: Review; published in the last 5 years* | ***192*** |
|  | (differentiated thyroid cancer OR Hurthle cell OR follicular thyroid cancer OR radioiodine-refractory thyroid cancer OR papillary thyroid cancer) AND (“treatment pattern” OR “treatment patterns” OR  management OR "treatment practice" OR "therapeutic approach") AND survival  *Limits: Review; published in the last 5 years* | ***54*** |
|  | **Total articles selected** | ***12*** |
|  |  |  |
| **Topic. Efficacy, safety, and patient-reported outcomes** |  |  |
|  | (differentiated thyroid cancer OR Hurthle cell OR follicular thyroid cancer OR radioiodine-refractory thyroid cancer OR papillary thyroid cancer) AND (treatment OR therapy)  *Limits: Clinical trial, Meta-analysis; published in the last 10 years* | ***344*** |
|  | *Lenvatinib OR E7080* | ***65*** |
|  | *1 OR 2* | ***408*** |
|  | **Total articles selected** | **2** |
|  |  |  |
| **Topic. Context and Implementation** |  |  |
|  | (thyroid cancer and occupation) | **49** |
|  | (rare disease plans and Europe OR EUROPLAN) | **32** |
|  | **Total articles selected** | **4** |

For the economic burden of disease, PubMed/Medline searches were conducted and additionally conference abstracts were searched, including the ESMO, ASCO, and ISPOR conferences. The keywords were “differentiated thyroid cancer, economics, burden of illness, cost”. A total of 212 search results were obtained.

**Web sites**

Table: Web sites

| Country/ Organization | Website |
| --- | --- |
| **Clinical trial registry** | <https://clinicaltrials.gov> |
| **Cochrane reviews** | <http://community.cochrane.org/cochrane-reviews> |
| **European Thyroid Association** | <http://www.eurothyroid.com/guidelines/eta_guidelines.html> |
| **European Society of Endocrinology** | <http://www.ese-hormones.org/guidelines/> |
| **European RARECARE registry** | <http://www.rarecare.eu/> |
| **EUROPLAN -European Project for Rare Disease National Plans** | <http://www.europlanproject.eu/Content?folder=1&content=1> |
| **European Commission – Rare Diseases (EU)** | <http://ec.europa.eu/health/rare_diseases/policy/index_en.htm> |
| **Rare Disease Task Force (EU)** | <http://www.rdtf.org> |
| **European Organization for rare diseases (EU)** | <http://www.eurordis.org> |
| **France**  **Centre Léon-Bérard: Cancer, Environnement et Nutrition** | <http://www.cancer-environnement.fr/287-Thyroide.ce.aspx>  <http://www.cancer-environnement.fr/LinkClick.aspx?fileticket=1_GJNr8Eukg%3d&tabid=287&mid=1605>  <http://www.cancer-environnement.fr/LinkClick.aspx?fileticket=Gtvml8kuRtY%3D&tabid=287&mid=1605> |
| **France**  **Haute Autorité de Santé** | <http://www.has-sante.fr/portail/jcms/fc_1249588/en/accueil> |
| **France**  **CHU Reseau** | <http://www.reseau-chu.org/fileadmin/reseau-chu/anciens-articles/_new/maladies_rares.htm> |
| **Italy**  **Italian Association of Cancer Registries (AIRTUM)** | <http://www.registri-tumori.it/cms/> |
| **Italy**  **Società Italiana di Otorinolaringologia e Chirurgia Cervico-Facciale** | <http://www.sioechcf.it/?page_id=138> |
| **Spain**  **Granada cancer registry** | <http://cancergranada.org/en/index.cfm> |
| **Spain**  **Cris Foundation for Cancer Research** | <https://www.criscancer.org/en/why-cris.php?zona=1&seccion=2&apartado=5> |
| **Spain**  **Centro de Investigation Biomedica en Red de Enfermedades Raras** | <http://www.ciberer.es/> |
| **World**  **Globocan** | <http://globocan.iarc.fr/Default.aspx> |
| **World**  **Orphanet** | http://www.orpha.net/consor/cgi-bin/index.php |

## Selection criteria of sources of data reported in the Evidence Matrix

Disease impact and context of intervention

Peer reviewed articles and reviews, clinical practice guidelines, and information published by Health Technology Assessment agencies were included. Where many articles were available on one subject, recent studies with large representative populations, and recent reviews in prominent journals were preferentially selected. Epidemiology information was preferentially sourced from recent peer-reviewed studies; where these were not available, registry data, prominent disease associations and databases were consulted. Conference abstracts or company internal data/reports were included when no other information was available.

Clinical studies

Only key pivotal studies (Phase 3 and 4 randomized controlled trials [RCTs]; prospective studies) were included in the assessment (evidence table and evidence matrix). Phase 1 and 2 RCTs, studies with no pooled data, and case series were not included. Research abstracts were also excluded, except when no other sources of information were available for specific topics (e.g. health utilities). In this case, only one placebo-controlled phase 3 trial (SELECT) was available and there were no head-to-head trials. Data from SELECT was supplemented by data from the phase 3 placebo trial of the comparator sorafenib (DECISION) and by an indirect treatment comparison (Eisai, manuscript in preparation).

Comparator studies (sorafenib) were selected based on the quality, relevance and validity and availability of reporting of similar outcomes. In this case, only one trial for sorafenib for the treatment of patients with RRDTC was available.

Contextual criteria

Relevant peer-reviewed articles and literature reviews as well as information published by steering committees were included, along with information from pan-European and national/regional rare disease organizations. Information from country-specific health system-sponsored rare disease initiatives was also included. Additional data were taken from international and regional thyroid cancer research networks and patient organizations.

## Data extraction and validation

Clinical data from the study drug’s eligible trials were extracted into the evidence table template (Appendix 4), then summarized and presented in the Evidence Matrix. Outcomes selection was based on regulatory requirements for oncology trials (FDA and CHMP[53,54]); efficacy outcomes included were overall survival (gold standard for oncology trials), progression-free survival (acceptable primary endpoint), and best objective response (convincing measure of drug activity in terms of tumor response). Relevant safety outcomes were common adverse events (AEs; both treatment-related and all-cause), serious AEs, discontinuations and deaths.

## Currency conversion

For the economic burden of disease, cost data were adjusted to Euros using historical currency conversion rates from Yahoo Finance (<http://finance.yahoo.com/> ).
