# Supplementary material for: Appraising the holistic value of Lenvatinib for radio-iodine refractory differentiated thyroid cancer: A multi-country study applying pragmatic MCDA
Source: BMC Cancer. 2017 Apr 17;17:272. doi: 10.1186/s12885-017-3258-9 (PMC5393009; doi:10.1186/s12885-017-3258-9)
Supplement: Supplementary file 3 — MCDA evidence matrix for lenvatinib in the Italian context. (DOCX 49 kb) [file 12885_2017_3258_MOESM3_ESM.docx]

# Additional File 3: MCDA Evidence Matrix for lenvatinib in the Italian context

## Scoring the performance of Lenvatinib – quantitative MCDA core model

| PRODUCT DESCRIPTION | *Drug class / intervention category: Protein kinase inhibitors (ATC code: L01XE29) – Lenvatinib is a receptor tyrosine kinase (RTK) inhibitor that selectively inhibits the kinase activities of vascular endothelial growth factor receptors, in addition to other proangiogenic and oncogenic pathway-related RTKs including fibroblast growth factor receptors 1, 2, 3, and 4, the platelet derived growth factor receptors PDGFRα, KIT, and RET.*  *Indication (EMA, anticipated):* *For the treatment of adult patients with progressive, locally advanced or metastatic, differentiated (papillary/follicular/Hürthle cell) thyroid carcinoma, refractory to radioactive iodine.*  *Dosage/Administration:* *Provided as 4-mg and 10-mg hard capsules for oral administration. The recommended daily dose of lenvatinib is 24 mg taken once daily. The daily dose is to be modified as needed according to the dose/toxicity management plan.*  *Intervention duration:* *Treatment should continue as long as there is clinical benefit*  *Comparators: Sorafenib and best supportive care* |
| --- | --- |
| ECONOMIC BURDEN OF DISEASE* | *Economic burden of illness: Total thyroid cancer-related annual healthcare cost per patient with advanced or metastatic differentiated thyroid carcinoma (DTC) in the US: €8,081 (US$10,953, 2014 values).[1] Outpatient costs represented 45%, inpatient costs 39%, and pharmacy costs 12% of this total cost. Of these patients, only 2.3% received targeted thyroid cancer therapy and 6.8% chemotherapy. Among US patients with radioiodine refractory DTC who had received a RTK inhibitor, the total thyroid-cancer-related annual healthcare cost per patient was €71,817 (US$97,344, 2014 values)[2]* |

*Not considered a criterion that contributes to the value of an intervention but provides useful background information. The overall economic burden of the disease is a composite measure of disease severity, size of affected population and cost of current treatment which are all captured in 3 distinct criteria of the quantitative MCDA core model.

| **INSTRUCTIONS:**   - For each criterion, assign a **score** (or a range of scores to reflect your uncertainty) based on the evidence available. Comments may be provided. - In case of no data, assign a score or a range of scores reflecting how you usually deal with such situations.   ***Important note*:** score ranges will be transformed into a measure of uncertainty. |
| --- |

| Criteria | Synthesis of available data for product | Score and comments |
| --- | --- | --- |
| **Domain: DISEASE IMPACT** | | |
| ***Disease severity***  *How severe is the disease targeted by the intervention?* | - Differentiated thyroid cancer (DTC), comprises papillary, follicular and Hürthle cell carcinomas.[3,4] **Radioiodine-refractory DTC** **(RRDTC)** is generally defined as disease in which **at least one lesion is unable to take up radioiodine (^131^I) or progresses despite radioiodine** **therapy**.[5,6] - Thyroid cancer can occur at any age; peak incidence is 65–69 years.[7] Main risk factors are exposure to ionising radiation, family history and female gender. No specific risk factors for RRDTC have been identified.[8,9]   Impact of disease on life-expectancy   - Data from placebo arms of two Phase III RCTs of patients with progressive, predominantly metastatic RRDTC indicate 63% overall survival at 18 months from randomisation;^[10]^ median overall survival was 19.1 months from randomisation.^[11,12]^ - In a retrospective study of patients with papillary or follicular thyroid carcinoma, survival 10 years after detection of metastasis was 10% among patients with lesion(s) unable to take up ^131^I, and 29% among patients who showed no remission following therapy with ^131^I.[13]   Impact of disease on morbidity   - Initial symptoms of RRDTC include a lump or swelling in the neck, dyspnoea, dysphagia, and hoarseness.[14] Increasing tumour burden with progressive RRDTC can cause severe symptoms due to airway obstruction, including pain, dysphagia, haemoptysis, hoarseness, and dyspnoea leading to asphyxia.[14,15] - Metastasis most commonly occurs in the lungs; other sites frequently affected are the lymph nodes, bone, head and neck, pleura, and liver.[11] Pain and organ dysfunction can occur with metastasis to distant sites.[15] - In a retrospective chart review of RRDTC patients receiving systemic treatments, bone pain was the most common symptom (reported by 30.7%).[16] Patients were hospitalised an average of 0.25 times for disease-related reasons in the last 12 months for an average of 7.5 days.[16]   Impact of disease on quality of life: Members of the UK general public (n=100) rated the utility of RRDTC states based on descriptions (vignettes) of patients’ health states: [17]   - Time-trade-off mean health utility (range 0=death, to 1=perfect health): stable disease, 0.80; progressive disease, 0.50 - Visual Analog Scale mean score (range 0=death, to 100=perfect health): stable disease, 57; progressive disease, 31 | **5 Very severe**  **4**  **3**  **2**  **1**  0 Not severe  Comments: |

| ***Size of affected population***  *What is the size of the population targeted by the intervention?* | DTC account for approximately 95% of all cancers of the thyroid.[4] The proportion of diagnosed DTC cases that become RRDTC is estimated at 2.5% based on data from the Marne-Ardennes Thyroid Registry, France, from 1983 to 2005.[18] Only a proportion of RRTDC patients are candidates for systematic therapy.   - Estimated prevalence of metastatic RRDTC in Italy: 3.8/100,000, based on 5-year prevalence of thyroid cancer (GLOBOCAN[19]) and 6% estimated proportion of metastatic RRDTC (market research data)   Thyroid cancer annual incidence:   - Overall (Italy): 10.8/100,000 (2012)[20] - Females (Italy): 24/100,000; males (Italy): 8.1/100,000 (2005–2009)[21]   From the above, annual incidence of RRDTC may be estimated as follows:  Incidence of thyroid cancer × proportion of thyroid cancers that are DTC × proportion of DTC that become RRDTC  10.8 × 0.95 × 0.0245 = **0.3/100,000 population**  ***See additional data in* : Additional data – EPIDEMIOLOGY** | **X=Prevalence or incidence**  **5: X > 500/10,000**  **4: X < 500/10,000**  **3: X < 100/10,000**  **2: X <10/10,000**  **1: X <5/10,000 (rare)**  0: X < 2 in 100,000 (ultra rare)  *Based on EMA definition*  Comments: |
| --- | --- | --- |
| **Domain: CONTEXT OF INTERVENTION** | | |
| ***Expert consensus/clinical practice guidelines***  *Is the product (or product of the same class) recommended in well-established guidelines? What type of recommendation (first line?, Level 1?)* | Excerpt from clinical practice guidelines specific for RRDTC  National Comprehensive Cancer Network (NCCN) 2.2014:[14,22] (all recommendations evidence level 2A):   - No specific recommendations for lenvatinib (not FDA-approved at the time of publication) - For progressive and/or symptomatic disease, consider sorafenib. Other small molecular kinase inhibitors (not FDA approved) can be considered if clinical trials or other systemic therapies are not available or appropriate. - Consider resection of distant metastases and/or external beam radiotherapy (EBRT) to metastatic lesions if progressive and/or symptomatic. EBRT may also be considered for asymptomatic bone metastases at weight-bearing sites. - Watchful waiting may be appropriate in asymptomatic patients with indolent disease. Kinase inhibitor therapy may not be appropriate for patients with stable or slowly progressive indolent disease.   British Thyroid Association, 2014: [23]   - Sorafenib and lenvatinib are the targeted agents demonstrating the most clinical activity for RRDTC (no evidence level; these agents were not approved at the time of publication) - The principal indication for targeted treatments is radiologically progressive, symptomatic disease, refractory to conventional treatments (evidence level 4, grade D) - Use targeted therapies outside clinical trials after careful consideration of the balance between potential benefits and harm (evidence level 4, grade D).Targeted therapies should only be administered in cancer units that have experience in monitoring and managing adverse effects (evidence level 4, grade D).   European Society of Endocrine Surgeons (ESES), 2014: [24] No recommendations regarding lenvatinib or other multikinase inbitors (MKIs).  Spanish Society of Medical Oncology (SEOM), 2014: [25]   - Patients with locoregional disease who do not have ^131^I uptake: external beam radiotherapy (EBRT) (category 2B) - Patients with locoregional or metastatic thyroid cancer refractory to ^131^I, who exhibit disease progression by “Response Evaluation Criteria In Solid Tumours” or who become symptomatic: sorafenib (category 1) or lenvatinib (category 1) or clinical trial or best supportive care   European Society for Medical Oncology (ESMO), 2012: [26] No recommendations for lenvatinib. MKIs to be administered only in clinical trials.  French ENT & Head Neck Surgery Society (Société Française d’Oto-Rhino-Laryngologie et de Chirurgie de la Face et du Cou (SFORL)), 2012: [27] No recommendations regarding lenvatinib or other MKIs.  French National Authority for Health (Haute Autorité de santé), 2010: [28] Chemotherapy can be discussed for the treatment of progressive disease refractory to conventional treatment, optionally in combination with targeted therapies (MKIs or HSP-90 inhibitors; in Phase II trials at the time of publication). | **5 Strong first-line recommendation for drug above all other alternatives**  **4**  **3**  **2**  **1**  **0 No recommendation or not recommended**  Comments: |
| ***Unmet needs***  *Are there many unmet needs to manage this disease with regard to the outcomes of comparative alternative interventions?* | Cytotoxic systematic chemotherapy, such as doxorubicin, has minimal efficacy in patients with metastatic disease.[14] Palliation of isolated skeletal metastases can be achieved with external beam radiation or surgical excision.[14] Bisphosphonates may help prevent skeletal adverse events. Brain metastases may respond to neurosurgical resection or stereotactic radiosurgery.[14]  ---------------------------------------------------------------------------------------------------------------------------------------------------------------------------------------------  Oral sorafenib is currently the only alternative treatment indicated for patients with progressive RRDTC. Its limitations include:   - Efficacy: In a phase III RCT, 12.2% of patients showed a response to sorafenib therapy and the median duration of response was 10.2 months.[11] PFS was prolonged by a median of 5.0 months compared to placebo (10.8 vs 5.8 months, *P*<.0001).[11] Effect on overall survival is uncertain: while ITT analyses show no statically significant differences in survival,[11,29] analyses that correct for placebo patients’ cross-over to sorafenib after disease progression (71% of placebo patients) indicate that sorafenib may provide a survival benefit.[30] - Safety / tolerability: The most important serious adverse reactions of sorafenib therapy are myocardial infarction (≥1%)/ischaemia, gastrointestinal perforation (≥0.1%), drug induced hepatitis (≥0.01%), haemorrhage (≥10%), and hypertension (≥10%)/hypertensive crisis (≥0.1%).[29] The most common (≥10% each) adverse reactions are diarrhoea, fatigue, alopecia, infection hand foot skin reaction and rash.[29] - Health-related quality of life (HRQL): Sorafenib therapy showed a small but statistically significant negative impact on both generic (EQ-5D) and specific (FACT-G) measures of HRQoL.[30] - Other: While TSH levels should be suppressed to <0.1 U/mL in patients with metastatic disease,[26] sorafenib therapy may increase TSH to >0.5 U/mL necessitating frequent TSH monitoring and adjustment in levothyroxine dose.[11,29] | **Scenario 1: Sorafenib NOT available**  **5 Many & serious**  **4**  **3**  **2**  **1**  0 No limitations  **Scenario 2: Sorafenib available**  **5 Many & serious**  **4**  **3**  **2**  **1**  0 No limitations  Comments: |
| **Domain: COMPARATIVE OUTCOMES OF INTERVENTION (EXTENT OF BENEFIT)**  **– see detailed Evidence Table in** **: Evidence tables** | | |
| ***Comparative effectiveness***  *How does this intervention compare to alternatives with respect to efficacy / effectiveness outcomes?* | **Efficacy data: *one pivotal phase 3 trial: SELECT:*** Randomized, double-blind, placebo-controlled, multicentre; progressive, locally advanced or metastatic (lung 89%; bone 39%) RRDTC; 96% ECOG 0-1; 61-64 yrs (median); only one previous tyrosine kinase inhibitor allowed.  Additional information: a global study (not started) will be conducted to evaluate the efficacy and safety of a lower (< 24 mg once daily) lenvatinib starting dosage.  **Comparators included in evaluation:**   - **Best supportive care:** placebo arm of SELECT trial (thyroid hormone suppression therapy received). - **Sorafenib: no head-to-head trials available; one *pivotal phase 3 trial*** ***DECISION*** double-blind placebo controlled; progressive locally advanced or metastatic (lung 86%; bone 27%) RRDTC; 97% ECOG 0-1; 63 yrs (median); no prior targeted therapy allowed; ***Indirect treatment comparison*** of lenvatinib versus sorafenib (EISAI, *manuscript in preparation*)  \| Data/Outcomes \| SELECT trial[10,31] \| \| DECISION trial[11,29,30] \| \| Indirect comparison \| \| --- \| --- \| --- \| --- \| --- \| --- \| \|  \| Lenvatinib  24 mg/day \| Placebo \| Sorafenib  400 mg bid \| Placebo \| Lenvatinib/  sorafenib \| \| N \| 261 \| 131 \| 207 \| 210 \| Risk Ratio (95% CI) \| \| Treatment duration, median \| 13.8 mo \| 3.9 mo \| 10.6 mo \| 6.5 mo \| \| Follow-up duration, median \| 17.1 mo  (1^st^ dataset) \| 17.4 mo \| 16.2 mo \| \| \| *Primary (ITT): Progression-free survival*  *median (range) mo* \| 18.3*mo  (15.1–NE) \| 3.6 mo  (2.2–3.7) \| 10.8* \| 5.8 \| 0.38  (0.24–0.58) \| \| *HR (95% CI); P value* \| 0.21 (0.14–0.31); *P*< .001* \| \| 0.59 (0.45–0.76); *P*< .0001* \| \|  \| \| *Overall survival (ITT), median (95% CI)†*  *24 mo (lenvatinib 1^st^dataset)* \| Non estimable (22.0–NE) \| Non estimable  (14.3–NE) \| Non estimable \| Non estimable \| *Not available* \| \| *HR (95% CI); P value* \| 0.62 (0.40–1.00); *P=*.05 \| \|  \| \| *34 mo (lenvatinib 2^nd^ dataset)* \| Non estimable*  (30.9–NE) \| 19.1  (14.3–NE) \| 0.80 (0.54–1.19); *P=*.14** \| \| 0.66  (0.36–1.19) \| \| *HR (95% CI); P value* \| 0.53 (0.34–0.82); *P*< .0051* \| \|  \| \| *Disease Control Rate  (complete + partial response + stable disease)* \| 87.7%* \| 55.7% \| 54.1%* \| 33.8% \| 0.98  (0.74–1.31) \| \| *Odds ratio (95% CI)* \| 5.05 (2.98–8.54); *P*< .001* \| \| *P*< .0001* \| \|  \| \| *Clinical Benefit Rate (complete + partial response + stable disease ≥ 23 weeks)* \| 80.1%* \| 31.3% \|  \|  \| -- \| \| *Odds ratio (95% CI)* \| 7.63 (4.55–12.79); *P*< .001* \| \| Not reported \| \| \| *Objective Response Rate (ORR, complete + partial response)* \| 64.8%* \| 1.5% \| 12.2%* \| 0.5% \| 1.72  (0.15–19.40) \| \| *Complete response*  *Partial response*  *Stable disease*  *Progressive disease*  *ORR duration, median (95%CI) mo*  *Time to response, median (95% CI) mo* \| 1.5%  63.2%  23.0%  6.9%  NR (16.8–NR)  2.0 (1.9–3.5) \| 0%  1.5%  54.2%  39.7%  --  5.6 (1.8–9.4) \| 0  12.2%*  --  --  10.2  -- \| 0  0.5%  --  --  --  -- \| -- \|   CI: confidence interval; HR: hazard ratio; ITT: intent-to-treat; mo: month; NE: non estimable; NR: not reached; * Statistical significance versus placebo; †adjusted for cross-over, using rank-preserving structural failure time (RPSFT). **Source pour les valeurs des essais cliniques : Sorafenib DECISION trial, Brose et al., Lancet 2014; 384 (9940): 319-328.  **Effictiveness data:** *Not available* | **Scenario 1: Sorafenib NOT available**  **5 Much better than placebo**  **4**  **3**  **2**  **1**  **0 No difference**  **-1**  **-2**  **-3**  **-4**  **-5 Much worse than placebo**  **Scenario 2: Sorafenib available**  **5 Much better than sorafenib**  **4**  **3**  **2**  **1**  **0 No difference**  **-1**  **-2**  **-3**  **-4**  **-5 Much worse than sorafenib**  Comments |

| ***Comparative safety / tolerability***  *How does this intervention compare to alternatives with respect to safety outcomes?* | **safety data** – Data from pivotal trials of lenvatinib (SELECT) and sorafenib (DECISION), indirect treatment comparison   \|  \| SELECT trial[10] (follow-up: 17 months) \| \| \| \| DECISION trial[11] (follow-up: 11.6 months) \| \| \| \| \| --- \| --- \| --- \| --- \| --- \| --- \| --- \| --- \| --- \| \|  \| **Lenvatinib**  **N=261** \| \| **Placebo**  **N=131** \| \| **Sorafenib**  **N=207** \| \| **Placebo**  **N=209** \| \| \| ***Grade*** \| **Any** \| **> 3** \| **Any** \| **> 3** \| **Any** \| **> 3** \| **Any** \| **> 3** \| \| **AEs, %** \| **97.3** \| **75.9** \| **59.5** \| **9.9** \| **98.6** \| **--** \| **87.6** \| **--** \| \| **Most common treatment-emergent AEs (occurring in > 30% patients), %** \|  \|  \|  \|  \|  \|  \|  \|  \| \| Hypertension \| 67.8 \| 41.8 \| 9.2 \| 2.3 \| 40.6 \| 9.7 \| 12.4 \| 2.4 \| \| Diarrhea \| 59.4 \| 8.0 \| 8.4 \| 0 \| 68.6 \| 5.3 \| 15.3 \| 1.0 \| \| Fatigue or asthenia \| 59.0 \| 9.2 \| 27.5 \| 2.3 \| 49.8 \| 5.3 \| 25.4 \| 1.4 \| \| Decreased appetite \| 50.2 \| 5.4 \| 11.5 \| 0 \| -- \| -- \| -- \| -- \| \| Decreased weight \| 46.4 \| 9.6 \| 9.2 \| 0 \| 46.9 \| 5.8 \| 13.9 \| 1.0 \| \| Nausea \| 41.0 \| 2.3 \| 13.7 \| 0.8 \| 20.8 \| 0 \| 11.5 \| 0 \| \| Stomatitis \| 35.6 \| 4.2 \| 3.8 \| 0 \| -- \| -- \| -- \| -- \| \| Palmar–plantar erythrodysesthesia syndrome \| 31.8 \| 3.4 \| 0.8 \| 0 \| 76.3 \| 20.3 \| 9.6 \| 0 \| \| Proteinuria \| 31.0 \| 10.0 \| 1.5 \| 0 \| -- \| -- \| -- \| -- \| \| Alopecia \| 11.1 \| 0 \| 3.8 \| 0 \| 67.1 \| -- \| 7.7 \| -- \| \| Rash/desquamation \| 16.1 \| 0.4 \| 1.5 \| 0 \| 50.2 \| 4.8 \| 11.5 \| 0 \| \| Anorexia \| -- \| -- \| -- \| -- \| 31.9 \| 2.4 \| 4.8 \| 0 \| \| **Discontinuation due to AEs, %** \| **14.2** \|  \| **2.3** \|  \| **18.8** \|  \| **3.8** \|  \| \| **Any serious AEs, %** \| **49.8** \|  \| **22.9** \|  \| **37.2** \|  \| **26.3** \|  \| \| **Treatment-related serious AEs, %** \| **30.3** \|  \| **6.0** \|  \| *--* \|  \| *--* \|  \| \| **Most common serious AEs (occurring in > 2% patients), %** \|  \|  \|  \|  \|  \|  \|  \|  \| \| Hypertension \| 3.4† \|  \| 0† \|  \| -- \|  \| -- \|  \| \| Pneumonia \| 2.3† \|  \| 0† \|  \| -- \|  \| -- \|  \| \| Secondary malignancy \| -- \|  \| -- \|  \| 4.3 \|  \| 1.9 \|  \| \| Dyspnea \| -- \|  \| -- \|  \| 3.4 \|  \| 2.9 \|  \| \| Pleural effusion \| -- \|  \| -- \|  \| 2.9 \|  \| 1.9 \|  \| \| **Fatal treatment-related AEs, %** \| **2.3*** \|  \| **0** \|  \| **0.4** \|  \| **0.4** \|  \|   AE: adverse event; CI: confidence interval; *1 case each of pulmonary embolism, haemorrhagic stroke, and general deterioration of physical health; †treatment-related serious AEs  **Indirect treatment comparison**: risk ratio of lenvatinib/sorafenib for serious AEs 1.52 (95% CI: 0.98–2.36); RR for discontinuation due to AEs: 0.73 (95% CI: 0.24–2.21) (EISAI, *manuscript in preparation*)  **WARNINGS**  ***Lenvatinib***: hypertension; proteinurea; renal failure and impairment; cardiac failure; hepatotoxicity; haemorrhage; GI perforation; impairment of TSH suppression.[32]  ***Sorafenib***: hypersensitivity; haemorrhage; hypocalcemia; TSH levels changes; dermatological toxicity; hypertension; cardiac ischemia/infarction; GI perforation; hepatic impairment.[33]  **MONITORING**  ***Lenvatinib***: regularly monitor blood pressure, urine protein, clinical symptoms or signs of cardiac decompensation, liver function, electrolyte abnormalities, and TSH levels.[32]  ***Sorafenib***: regularly monitor blood pressure, electrocardiograms and electrolytes, liver function, and TSH levels.[33] | **Scenario 1: Sorafenib NOT available**  **5 Much better than placebo**  **4**  **3**  **2**  **1**  **0 No difference**  **-1**  **-2**  **-3**  **-4**  **-5 Much worse than placebo**  **Scenario 2: Sorafenib available**  **5 Much better than sorafenib**  **4**  **3**  **2**  **1**  **0 No difference**  **-1**  **-2**  **-3**  **-4**  **-5 Much worse than sorafenib**  Comments |
| --- | --- | --- | --- | --- | --- | --- | --- | --- | --- | --- | --- | --- | --- | --- | --- | --- | --- | --- | --- | --- | --- | --- | --- | --- | --- | --- | --- | --- | --- | --- | --- | --- | --- | --- | --- | --- | --- | --- | --- | --- | --- | --- | --- | --- | --- | --- | --- | --- | --- | --- | --- | --- | --- | --- | --- | --- | --- | --- | --- | --- | --- | --- | --- | --- | --- | --- | --- | --- | --- | --- | --- | --- | --- | --- | --- | --- | --- | --- | --- | --- | --- | --- | --- | --- | --- | --- | --- | --- | --- | --- | --- | --- | --- | --- | --- | --- | --- | --- | --- | --- | --- | --- | --- | --- | --- | --- | --- | --- | --- | --- | --- | --- | --- | --- | --- | --- | --- | --- | --- | --- | --- | --- | --- | --- | --- | --- | --- | --- | --- | --- | --- | --- | --- | --- | --- | --- | --- | --- | --- | --- | --- | --- | --- | --- | --- | --- | --- | --- | --- | --- | --- | --- | --- | --- | --- | --- | --- | --- | --- | --- | --- | --- | --- | --- | --- | --- | --- | --- | --- | --- | --- | --- | --- | --- | --- | --- | --- | --- | --- | --- | --- | --- | --- | --- | --- | --- | --- | --- | --- | --- | --- | --- | --- | --- | --- | --- | --- | --- | --- | --- | --- | --- | --- | --- | --- | --- | --- | --- | --- | --- | --- | --- | --- | --- | --- | --- | --- | --- | --- | --- | --- | --- | --- | --- | --- | --- | --- | --- | --- | --- | --- | --- | --- | --- | --- | --- | --- | --- | --- | --- | --- | --- | --- | --- | --- |

| ***Comparative patient-perceived health/ patient-reported outcomes***  *How does this intervention compare to alternatives with respect to patient-perceived health / patient-reported outcomes?* | **PATIENT-REPORTED OUTCOMES (PRO)/QUALITY OF LIFE (QOL) DATA**  ***Lenvatinib*** – SELECT trial: PRO/QoL data not collected.  ***Sorafenib*** – DECISION trial: small but statistically significant deterioration of health-related quality of life versus placebo[30]   - EQ-5D index and VAS scores: 0.69 vs 0.76 placebo and 67.62 vs 73.71 placebo, respectively; *P* < .0001 mixed linear model analysis - FACT-G scores functional well-being and total scores: treatment effect for FACT-G total score is -3.45 (95% CI: -5.41,-1.49), *P* < .0006 (mixed linear model analysis).   ***RRDTC health utilities*** – estimates of UK societal health-related utility value (range 0-1) for RRDTC health states (defined by RECIST criteria), effect of treatment response and most relevant toxicities/AEs associated with tyrosine kinase inhibitors[17]   - Deterioration (decrease) due to AEs and disease progression - Modest improvement (increase) upon response to treatment  \| **Parameter** \| **Utility value** \| **95% CI** \| \| --- \| --- \| --- \| \| Base state – Stable/no response \| 0.87 \| 0.84 / 0.91 \| \| Response to therapy \| +0.04 \| 0.01 / 0.07 \| \| Progressive disease \| -0.35 \| -0.41 / -0.29 \| \| Diarrhoea \| -0.47 \| -0.523 / -0.41 \| \| Fatigue \| -0.08 \| -0.12 / 0.04 \| \| Hand and foot syndrome \| -0.34 \| -0.40 / 0.28 \| \| Alopecia \| -0.05 \| -0.08 / 0.01 \|   **CONVENIENCE**  Lenvatinib (and its comparator sorafenib) is administered orally once daily. | **Scenario 1: Sorafenib NOT available**  **5 Much better than placebo**  **4**  **3**  **2**  **1**  **0 No difference**  **-1**  **-2**  **-3**  **-4**  **-5 Much worse than placebo**    **Scenario 2: Sorafenib available**  **5 Much better than sorafenib**  **4**  **3**  **2**  **1**  **0 No difference**  **-1**  **-2**  **-3**  **-4**  -5 Much worse than sorafenib  Comments |
| --- | --- | --- | --- | --- | --- | --- | --- | --- | --- | --- | --- | --- | --- | --- | --- | --- | --- | --- | --- | --- | --- | --- | --- | --- | --- | --- |
| **Domain: TYPE OF HEALTH BENEFIT OF INTERVENTION** | | |
| ***Type of preventive benefit***  *What type of preventative health gain or reduction of risk of disease is provided by the intervention?* | Lenvatinib does not prevent or modify the risk of developing RRDTC. | **5 Eradication / major risk reduction**  **4**  **3**  **2**  **1**  **0 No reduction in risk of disease**  Comments |
| ***Type of therapeutic benefit***  *What type of health gain is provided by the intervention?* | Lenvatinib does not cure RRDTC. It delays disease progression and there is data to support a prolongation of survival. | **5 Cure / life saving**  **4**  **3**  **2**  **1**  **0 No impact on existing condition**  Comments |
| **Domain: ECONOMIC CONSEQUENCES OF INTERVENTION** | | |
| ***Comparative cost consequences – cost of intervention***  *What is the direct cost impact of the intervention (including acquisition and administration costs?* | - **Intervention price, frequency and/or duration of administration and cost of administration** (based on manufacturer’s budget impact model) **— *Note: The lenvatinib prices shown represent a potential estimated price range.***  \|  \| **Lenvatinib** \| **Sorafenib** \| **Chemotherapy*** \| \| --- \| --- \| --- \| --- \| \| **Price per available dosing form** \| 4-mg or 10-mg capsule: €53.99–67.49 \| 200-mg capsule: €31.86 \| NA \| \| **Recommended daily dose (SmPC)** \| 24 mg/day \| 800 mg/day \| NA \| \| **Actual daily dose †** \| 16.6 mg/day \| 689 mg/day \| NA \| \| **Average daily drug cost per patient (based on actual dose)** \| €113 – €141 \| €110 \| €3 \| \| **Average monthly drug cost per patient** \| €3,428 – €4,285 \| €3,341 \| €96 \| \| **Administration cost per month‡** \| €387 \| €387 \| €860 \| \| **Average total monthly cost per patient** \| €3,815 – €4,672 \| €3,728 \| €956 \| \| **Average duration of treatment §** \| 18.3 months \| 10.8 months \| 12 months \| \| **Average total cost per treatment course** \| €69,815 – €85,498 \| €40,262 \| €11,472 \|   NA: not applicable  Assumptions and sources:  * All patients assumed to be treated with doxorubicin.  †Lenvatinib: Patients assumed to receive recommended dose (24 mg/d) for 2.76 months and then reduce dose to mean last dose in phase III (SELECT) trial (15.3 mg/d); sorafenib: Patients assumed to receive recommended dose (800 mg/d) for 2.76 months and then reduce dose to mean dose in phase III (DECISION trial) (651 mg/d)[11]  ‡Lenvatinib and sorafenib: includes required monthly laboratory tests;[34] chemotherapy: includes IV administration costs[34]  ^§^Lenvatinib and sorafenib: based on progression-free survival (PFS) duration in phase III studies (SELECT and DECISION);[10,11] chemotherapy: assumption   - **Annual projected impact of reimbursing lenvatinib on Italian drug spending for RRDTC** (includes drug and administration costs, based on manufacturer’s epidemiological budget impact model)   **Scenario 1. Sorafenib NOT available: Compare Years 1–5 with chemotherapy only (status quo) versus Years 1–5 with chemotherapy plus lenvatinib**   \|  \| **Number of patients treated with lenvatinib** \| **Average annual total* cost per patient treated with lenvatinib†** \| **Total* annual cost of lenvatinib** \| **Incremental (net) impact of lenvatinib on total* RRDTC drug spending** \| \| --- \| --- \| --- \| --- \| --- \| \| Year 1 \| 666 \| €45,774 – €56,059 \| €30.47 – €37.32 million \| €23.10 – €29.95 million \| \| Year 2 \| 402 \| €80,575 – €99,811 \| €32.36 – €40.08 million \| €27.98 – €35.71 million \| \| Year 3 \| 278 \| €76,057 – €94,131 \| €21.17 – €26.20 million \| €18.22 – €23.25 million \| \| Year 4 \| 221 \| €72,222 – €89,310 \| €15.95 – €19.73 million \| €13.66 – €17.43 million \| \| Year 5 \| 194 \| €69,656 – €86,083 \| €13.53 – €16.72 million \| €11.54 – €14.73 million \| \| **Total 5 years** \| **1,761** \| **€64,450 – €79,539** \| **€113.48 – €140.05 million** \| **€94.50 – €121.07 million** \|   *Total includes drug costs and administration costs; †Calculation takes into account that lenvatinib’s treatment course is longer than 12 months.  Assumptions and sources:   - - - **Number of patients:** Prevalence of metastatic RRDTC in Italy: 1,861 patients (=3.68/100,000 adult population) including 253 new (incident) cases annually (=0.50/100,000) (sources: GLOBOCAN[19] and market research data); 67% of these patients (incident and prevalent) receive chemotherapy or MKI (approx. 1,247 patients = 1,077 prevalent + 170 incident) based in EU5 market research data.     - **Market share and switching:** Year 0: All treated patients receive chemotherapy. Years 1-5: ***If no lenvatinib available***: All treated patients receive chemotherapy. ***If lenvatinib available***: 100% of new (incident) patients receive lenvatinib, and each year 46% of prevalent patients switch from chemotherapy to lenvatinib. *Note:* Patients can only receive one course of lenvatinib but multiple courses of chemotherapy. These assumptions are based on internal projections and US market research data.   **Sensitivity analysis**: 5-year incremental budget impact reduces to €73.09 – €94.14 million if treatment duration is reduced to median treatment duration in lenvatinib phase III clinical trial (13.8 months).  **Scenario 2. Sorafenib available: Compare Years 1–5 with chemotherapy & sorafenib versus Years 1–5 with chemotherapy & sorafenib plus lenvatinib**   \|  \| **Number of patients treated with lenvatinib** \| **Average annual total* cost per patient treated with lenvatinib†** \| **Total* annual cost of lenvatinib** \| **Incremental (net) impact of lenvatinib on total* RRDTC drug spending** \| \| --- \| --- \| --- \| --- \| --- \| \| Year 1 \| 266 \| €45,774 – €56,059 \| €12.19 – €14.93 million \| €1.43 – €4.17 million \| \| Year 2 \| 402 \| €59,671 – €73,531 \| €24.00 – €29.54 million \| €17.80 – €23.38 million \| \| Year 3 \| 320 \| €72,184 – €89,262 \| €23.08 – €28.54 million \| €19.47 – €24.93 million \| \| Year 4 \| 243 \| €73,346 – €90,723 \| €17.85 – €22.08 million \| €15.31 – €19.54 million \| \| Year 5 \| 198 \| €71,574 – €88,494 \| €14.17 – €17.52 million \| €11.98 – €15.33 million \| \| **Total 5 years** \| **1,430** \| **€63,858 – €78,794** \| **€91.29 – €112.65 million** \| **€65.99 – €87.35 million** \|   *Total includes drug costs and administration costs; †Calculation takes into account that lenvatinib’s treatment course is longer than 12 months.  Assumptions and sources:   - - - **Number of patients:** as above     - **Market share and switching:** Year 0: as above. Years 1–5: ***If no lenvatinib available***: 100% of new (incident) patients receive sorafenib, and each year 46% of prevalent patients switch from chemotherapy to sorafenib. ***If lenvatinib available***: 40% of new patients receive lenvatinib and 60% sorafenib, and each year 18.4% of prevalent chemotherapy patients switch to lenvatinib and 27.6% to sorafenib. After sorafenib failure, 70% switch to lenvatinib and 30% to chemotherapy; after lenvatinib failure, 46% switch to sorafenib and 54% to chemotherapy. *Note:* Patients can only receive one course of lenvatinib and sorafenib but multiple courses of chemotherapy. Source of assumptions as above.   **Sensitivity analysis**: 5-year incremental budget impact reduces to €49.45 – €66.48 million if treatment duration is reduced to median treatment duration in phase III clinical trials (lenvatinib 13.8 months and sorafenib 10.6 months). | **Scenario 1: Sorafenib NOT available**  **5 Substantial savings**  **4**  **3**  **2**  **1**  **0 No difference**  **-1**  **-2**  **-3**  **-4**  **-5 Substantial additional expenditures**  **Scenario 2: Sorafenib available**  **5 Substantial savings**  **4**  **3**  **2**  **1**  **0 No difference**  **-1**  **-2**  **-3**  **-4**  **-5 Substantial additional expenditures**  Comments |
| ***Comparative cost consequences – other costs***  *What is the impact of the intervention on other spending such as hospitalization, visits, tests, long term care, productivity?* | **Source:** Manufacturer’s epidemiological budget impact model  **Methods**   - **Progression-free survival**: for lenvatinib (18.3 months) and sorafenib (10.8 months) based on phase III (SELECT and DECISION) trials;[10,11] for chemotherapy (3.6 months) based on placebo arm of phase III SELECT trial.[11] The amount of time spent in PFS impacts the medical costs (physician visits and disease associated hospital days). - **RRDTC-related resource utilization**: See table below, data from EU5 RRDTC chart audit, 2014 (*manuscript in preparation*)  \| **Type of resource** \| **Average annual resource use per patient with** \| \| \| \| --- \| --- \| --- \| --- \| \| **Response** \| **Stable disease** \| **Progressive disease** \| \| Physician visits \| 11.5 \| 9.5 \| 12.9 \| \| Disease-associated hospitalization days \| 1.1 \| 1.2 \| 7.9 \|  - **Adverse events:** Unit costs from Ministero della Salute and EUMed.[35,36] Includes grade 3–4 AEs: hypertension (€1,669*), weight decrease (€55†), diarrhoea (€1,669*), decreased appetite (€55†), hypocalcaemia (€1,669*), hypokalaemia (€1,669*), asthenia (€55†), fatigue (€55†), hand-foot skin reaction (€1,669*), and proteinuria (€1,669*). Frequency based on phase III (SELECT and DECISION) trials for lenvatinib and sorafenib.[10,11] Due to lack of phase III trial data for chemotherapy AE frequencies assumed to be the same as for sorafenib.  * Includes 2 physician visits plus 5 days of hospitalisation † Includes 2 physician visits.   **Results**  **Scenario 1. Sorafenib NOT available: Compare Years 1–5 with chemotherapy only (status quo) versus Years 1–5 with chemotherapy plus lenvatinib**   \|  \| **Average annual cost per patient treated with** \| \| \| --- \| --- \| --- \| \| **Lenvatinib** \| **Chemotherapy** \| \| RRDTC-related costs \| €937 \| €2,838 \| \| Adverse event costs \| €155 \| €87 \|  - Adoption of lenvatinib is projected to save €1.87 million over 5 years using the market share and switching assumptions reported in section **5.5.1** above.   **Scenario 2. Sorafenib available: Compare Years 1–5 with chemotherapy & sorafenib versus Years 1–5 with chemotherapy & sorafenib plus lenvatinib**   \|  \| **Average annual cost per patient treated with** \| \| \| \| --- \| --- \| --- \| --- \| \| **Lenvatinib** \| **Sorafenib** \| **Chemotherapy** \| \| RRDTC-related costs \| €928 \| €769 \| €3,044 \| \| Adverse event costs \| €155 \| €68 \| €93 \|  - Adoption of lenvatinib is projected to save €1.28 million over 5 years using the market share and switching assumptions reported in section **5.5.1** above. | **Scenario 1: Sorafenib NOT available**  **5 Substantial savings**  **4**  **3**  **2**  **1**  **0 No difference**  **-1**  **-2**  **-3**  **-4**  **-5 Substantial additional expenditures**  **Scenario 2: Sorafenib available**  **5 Substantial savings**  **4**  **3**  **2**  **1**  **0 No difference**  **-1**  **-2**  **-3**  **-4**  **-5 Substantial additional expenditures**  Comments |
| **Domain: QUALITY / UNCERTAINTY OF EVIDENCE**  **– see detailed assessment of quality in : Analysis of the Quality of Evidence** | | |
| ***Quality of evidence***  *What is the quality of the design of studies and their relevance to the context?* | **Assessment of the clinical program**  The clinical program of lenvatinib for the treatment of progressive RR-DTC includes a single-arm phase 2 trial (excluded from this assessment focusing on best available data) and a phase 3 randomized placebo-controlled trial (SELECT trial; included in this assessment). Supportive information is provided by an indirect treatment comparison versus sorafenib (the only tyrosine kinase inhibitor approved for progressive RRDTC).  The SELECT trial is well designed and relevant in terms of target population, size, time horizon, and efficacy/safety outcomes selection. Patient-relevant outcomes (patient-reported outcomes, quality of life), however, were not assessed in this trial. Results of secondary endpoints (overall survival [78% at 24 months] and response rate [50%]) are consistent with the phase 2 trial results.[37] | **5 Well reported, highly relevant and valid**  **4**  **3**  **2**  **1**  **0 Not relevant and/or invalid**  Comments |

## Considering contextual criteria – Qualitative MCDA Contextual Tool

| **INSTRUCTIONS:**   - Based on evidence and your insights, indicate how the consideration of each criterion **impacts** your appraisal of the intervention in the context of your healthcare system. - You may provide your insights/colloquial evidence relative to the criteria (optional). - Comments may be provided. |
| --- |

| Criteria | Synthesis of available data for product | Score and comments |
| --- | --- | --- |
| **CONTEXT AND IMPLEMENTATION** | | |
| ***Mandate and scope of healthcare system*** | *Does lenvatinib’s use in RRDTC align with the mandate and scope of your country’s healthcare system?*  *Does the consideration of this criterion have an impact on the value of the intervention (i.e., does lenvatinib’s status as a treatment for a rare disease impact the appraisal of its value)?*  **Evidence**: The treatment of advanced thyroid cancer aligns with the goals of healthcare systems in Italy, France, and Spain. The disease is both serious and life threatening; if untreated, patients with RRDTC face a significant disease burden and short survival.[6,15,33]  **Insights from appraiser**: | **Positive impact**  **No impact**  **Negative impact** |
| ***Population priorities & access (principle of fairness)*** | *How is lenvatinib for use in RRDTC aligned with the specifics of the established priority for rare diseases?*  *Are there other patient population prioritization factors to be considered?*  **Evidence:** Thyroid cancer affects several patient groups disproportionately, including women (aged 40-49) and also individuals of low socioeconomic status.[3] Available literature suggest potential links between thyroid cancer and occupation-related exposure to chemicals (e.g. pesticides[38] and industrial solvents[39]) and/or radiation, meaning that those who work in certain industries may be at greater risk for developing thyroid cancer.[38]  RRDTC meets EMA’s definition for an orphan disease and lenvatinib has been granted orphan drug designation. The EUROPLAN Project[40] and the EUCERD Joint Action have been established to assist EU member countries in building their own initiatives to support rare disease research and treatment.[41] At this time, France, Italy, Spain, and all have their own country-specific rare disease initiatives.[42-45]  In **Italy,** the Ministry of Health has established a national network for prevention, surveillance, diagnosis and treatment of rare diseases, a National Registry of Rare Diseases, and a waiver for medical care cost, in addition to a diagnostic work-up and therapy for patients with a suspicion or diagnosis of a rare disease included in its identified list. Further, the plan has established an inventory of rare conditions which receive specific cost exemption. As of 2008 the National Centre for Rare Diseases (CNMR) was established at ISS (the Italian National Institute for Health), with the mission of promoting and developing scientific research and public health actions, as well as providing technical expertise and information on rare diseases and orphan medicinal products, aimed at the prevention, treatment and surveillance of these diseases.[45]  **Insights from appraiser**: | **Positive impact**  **No impact**  **Negative impact**  **If priorities for the drug exist, by which percentage this should impact the quantitative value estimate established using the MCDA core model?**  **________%** |
| ***Opportunity costs & affordability*** | *Does lenvatinib results in significant displacement of resources of the healthcare system in your country/region?*  **Evidence:** Lenvatinib is anticipated to have a small budget impact due to the small number of RRDTC patients [13,46,47]. Lenvatinib’s use could lead to disinvestments in off-label therapies such as chemotherapy or other TKIs. However, it should be acknowledged that the cost per patient may be relatively high.  **Insights from appraiser**: | **Positive impact**  **No impact**  **Negative impact** |
| ***System capacity and appropriate use of intervention*** | *Does the healthcare system in your country have sufficient capacity (e.g., skills, knowledge of intervention, surveillance system) to ensure appropriate use of lenvatinib?*  *Does the consideration of this criterion have an impact on the value of the intervention?*  **Evidence:** Multikinase inhibitors are associated with potentially serious cardiac, renal, hepatic, hematologic, and other side effects which require careful monitoring and management.[48] Specifically, lenvatinib is associated with SAEs, including hypertension, cardiac failure, arterial thromboembolic events, hepatotoxicity, proteinuria, renal failure/impairment, GI perforation and fistula, haemorrhagic events, and others.[49] Patients must be monitored for these conditions using cardiac and laboratory diagnostics (and potentially others), and if present, treated appropriately.[49]  In addition, RRDTC itself can be difficult to detect and treatment is not indicated until there is evidence for progressive radioiodine refractory disease, which may require imaging tests such as neck ultrasound, chest CT, spine and brain MRI, bone scans, and Fluorodeoxyglucose (FDG) PET scans.[50] Beyond diagnosis, these tests will also be necessary for effective monitoring of treatment response and disease management.  **Insights from appraiser**: | **Positive impact**  **No impact**  **Negative impact** |
| ***Common goal and special interests*** | *Are you aware of pressures/barriers from stakeholders regarding lenvatinib?*  *Does the consideration of this criterion have an impact on the value of the intervention?*  **Evidence:** Some physicians and payers are opposed to giving special consideration to rare disease treatments, arguing instead for equity on the grounds that all medical treatments should be appraised on equal footing.[51] Conversely, patient advocacy groups (e.g. European Organization for Rare Diseases) and disease specific research organizations (e.g. European Thyroid Association Cancer Research Network) create pressure to raise rare disease issues on the agenda.  **Insights from appraiser**: | **Positive impact**  **No impact**  **Negative impact** |
| ***Political, historical, cultural context*** | *Are you aware of any political/historical factors which may impact the approval of lenvatinib (such as precedence, impact on innovation, impact on collaboration within the healthcare system)?*  *Does the consideration of this criterion have an impact on the value of the intervention?*  **Evidence:** Healthcare costs continue to rise along with increased pressure from national budget deficits. For instance, the European Steering Group on Sustainable Healthcare has released a white paper this year (2015) outlining a proposal for long-term cost control across EU health systems.[52]  **Insights from appraiser**: | **Positive impact**  **No impact**  **Negative impact** |
| ***Environmental impact*** | *Are you aware of environmental impact regarding lenvatinib?*  *Does the consideration of this criterion have an impact on the value of the intervention?*  **Evidence**: There is no information on the environmental impact of lenvatinib therapy**.**  **Insights from appraiser** | **Positive impact**  **No impact**  **Negative impact** |

## References

1 Hirst C, Kern DM, Zhou S, Tunceli O, Tchinou C, Prahladen M, Ryan J. Resource use associated with patients with thyroid cancer in a US insured population. Poster presented at ISPOR 19th Annual International Meeting; 2014 May 31 - June 4.; Montreal, QC, Canada.

2 Abouzaid S, Li X, Rietschel P. Health care utilization and cost associated with radioiodine refractory (RAI-R) Differentiated thyroid cancer (DTC). Abstract presented at ISPOR 20th Annual International Meeting; 2015 May 16-20.; Philadelphia.

3 Aschebrook-Kilfoy B, Ward MH, Sabra MM, Devesa SS. Thyroid cancer incidence patterns in the United States by histologic type, 1992-2006. Thyroid. 2011;21(2):125-34.

4 Hundahl SA, Cady B, Cunningham MP, Mazzaferri E, McKee RF, Rosai J, et al. Initial results from a prospective cohort study of 5583 cases of thyroid carcinoma treated in the united states during 1996. U.S. and German Thyroid Cancer Study Group. An American College of Surgeons Commission on Cancer Patient Care Evaluation study. Cancer. 2000;89(1):202-17.

5 Schlumberger M, Sherman SI. Approach to the patient with advanced differentiated thyroid cancer. Eur J Endocrinol. 2012;166(1):5-11.

6 Schlumberger M, Brose M, Elisei R, Leboulleux S, Luster M, Pitoia F, et al. Definition and management of radioactive iodine-refractory differentiated thyroid cancer. Lancet Diabetes Endocrinol. 2014;2(5):356-8.

7 Howlader N, Noone AM, Krapcho M, Garshell J, Miller D, Altekruse SF, et al., editors. SEER Cancer Statistics Review, 1975-2011. Section 26. Thyroid. On National Cancer Institute website. 2014. <http://seer.cancer.gov/csr/1975_2011/>. Accessed 2015 Mar 27.

8 Duntas L, Grab-Duntas BM. Risk and prognostic factors for differentiated thyroid cancer. Hell J Nucl Med. 2006;9(3):156-62.

9 Marcello MA, Malandrino P, Almeida JF, Martins MB, Cunha LL, Bufalo NE, et al. The influence of the environment on the development of thyroid tumors: a new appraisal. Endocr Relat Cancer. 2014;21(5):T235-T254.

10 Schlumberger M, Tahara M, Wirth LJ, Robinson B, Brose MS, Elisei R, et al. Lenvatinib versus placebo in radioiodine-refractory thyroid cancer. N Engl J Med. 2015;372(7):621-30.

11 Brose MS, Nutting CM, Jarzab B, Elisei R, Siena S, Bastholt L, et al. Sorafenib in radioactive iodine-refractory, locally advanced or metastatic differentiated thyroid cancer: a randomised, double-blind, phase 3 trial. Lancet. 2014;384(9940):319-28.

12 Eisai Inc. Indirect treatment comparison: lenvatinib versus sorafenib. Version 2.0 - New data cut. Figure D80.R103.303.2 Kaplan-Meier plot of overall survival adjusted with RPSFT model. Full analysis set. 2015 Feb 13.

13 Durante C, Haddy N, Baudin E, Leboulleux S, Hartl D, Travagli JP, et al. Long-term outcome of 444 patients with distant metastases from papillary and follicular thyroid carcinoma: benefits and limits of radioiodine therapy. J Clin Endocrinol Metab. 2006;91(8):2892-9.

14 National Comprehensive Cancer Network. NCCN clinical practice guidelines in oncology (NCCN guidelines). Thyroid carcinoma. Version 2.2014. On National Comprehensive Cancer Network website. 2014. https://[www.nccn.org/professionals/physician_gls/f_guidelines_nojava.asp](http://www.nccn.org/professionals/physician_gls/f_guidelines_nojava.asp). Accessed 2015 Mar 27.

15 Greenblatt DY, Chen H. Palliation of advanced thyroid malignancies. Surg Oncol. 2007;16(4):237-47.

16 Eisai. Treatment patterns and health outcomes among patients with radioiodine-refractory differentiated thyroid cancer. 2015 Mar 30.

17 Kerr C, Fordham B, de Freitas HM, Tremblay G, Johnston K. Health state valuation in radio-iodine refractory differentiated thyroid cancer (RR-DTC). Poster presented at ISPOR 17th Annual European Congress; 2014 Nov 8-12.; Amsterdam.

18 Schvartz C, Dalac A, Ancelle D, Pochart J, Fieffe S, Patey M. Epidemiology of refractory thyroid cancer. Thyroid. 2012;22A109-A110

19 International Agency for Research on Cancer (IARC). Spain: Estimated incidence and prevalence, adult population: both sexes. On GLOBOCAN website. 2012. <http://globocan.iarc.fr/old/summary_table_pop_prev.asp?selection=182724&title=Spain&sex=0&window=1&sort=0&submit=%C2%A0Execute%C2%A0>. Accessed 2015 Mar 27.

20 International Agency for Research on Cancer (IARC). Italy: Estimated incidence and prevalence, adult population: both sexes. On GLOBOCAN website. 2012. <http://globocan.iarc.fr/old/summary_table_pop_prev.asp?selection=95380&title=Italy&sex=0&window=1&sort=0&submit=%C2%A0Execute%C2%A0>. Accessed 2015 Mar 27.

21 Italian Association of Cancer Registries (AIRTUM). Thyroid. On Italian Association of Cancer Registries (AIRTUM) website. 2015. <http://www.registri-tumori.it>. Accessed 2015 Mar 20.

22 Tuttle RM, Haddad RI, Ball DW, Byrd D, Dickson P, Duh QY, et al. Thyroid carcinoma, version 2.2014. J Natl Compr Canc Netw. 2014;12(12):1671-80.

23 Perros P, Boelaert K, Colley S, Evans C, Evans RM, Gerrard BG, et al. Guidelines for the management of thyroid cancer. Clin Endocrinol (Oxf ). 2014;81 Suppl 1:1-122.

24 Dionigi G, Kraimps JL, Schmid KW, Hermann M, Sheu-Grabellus SY, De WP, et al. Minimally invasive follicular thyroid cancer (MIFTC)--a consensus report of the European Society of Endocrine Surgeons (ESES). Langenbecks Arch Surg. 2014;399(2):165-84.

25 Trigo JM, Capdevila J, Grande E, Grau J, Lianes P. Thyroid cancer: SEOM clinical guidelines. Clin Transl Oncol. 2014;16(12):1035-42.

26 Pacini F, Castagna MG, Brilli L, Pentheroudakis G. Thyroid cancer: ESMO Clinical Practice Guidelines for diagnosis, treatment and follow-up. Ann Oncol. 2012;23 Suppl 7:vii110-vii119.

27 Guerrier B, Berthet JP, Cartier C, Dehesdin D, Edet-Sanson A, Le CG, et al. French ENT Society (SFORL) practice guidelines for lymph-node management in adult differentiated thyroid carcinoma. Eur Ann Otorhinolaryngol Head Neck Dis. 2012;129(4):197-206.

28 Haute Autorité de Santé. ALD n° 30. Cancer de la thyroïde. On Haute Autorité de Santé website. 2010. <http://www.has-sante.fr/portail/jcms/c_969305/fr/ald-n-30-cancer-de-la-thyroide>. Accessed 2015 Mar 26.

29 European Medicines Agency. Product information. Nexavar. On European Medicines Agency website. 2015. <http://www.ema.europa.eu/ema/index.jsp?curl=pages/medicines/human/medicines/000690/human_med_000929.jsp&mid=WC0b01ac058001d124>. Accessed 2015 Mar 26.

30 European Network for Health Technology Assessment (EUnetHTA). Sorafenib for the treatment of progressive, locally advanced or metastatic, differentiated (papillary/follicular/Hurthle cell) thyroid carcinoma, refractory to radioactive iodine ID: SA-[3]. On European Network for Health Technology Assessment (EUnetHTA) website. 2015. <http://www.eunethta.eu/outputs/joint-assessment-sorafenib-nexavar-and-its-use-treatment-progressive-locally-advanced-or-met>.

31 European Medicines Agency. CHMP assessment report. Lenvima. 2015 Mar 26.

32 European Medicines Agency. Product characteristics. Lenvima. 2015.

33 Bayer and Onyx Pharmaceuticals. Prescribing information. Nexavar. 2013 Nov.

34 Schwander B, Ravera S, Giuliani G, Nuijten M, Walzer S. Cost comparison of second-line treatment options for late stage non-small-cell lung cancer: cost analysis for Italy. Clinicoecon Outcomes Res. 2012;4:237-43.

35 EUMed. Tariffa Unica Convenzionale per le Prestazioni di Assistenza Ospedaliera per Acuti 2006. On EUMed website. 2006. <http://www.ncbi.nlm.nih.gov/pmc/articles/PMC3381065/#CR43>. Accessed 2010 May.

36 Ministero della Salute. Tariffa euro in vigore da prenotazioni, Lombardia (unpublished). On Ministero della Salute website. 2008. <http://www.ncbi.nlm.nih.gov/pmc/articles/PMC3381065/#CR35>. Accessed 2015 Apr 16.

37 Eisai Inc. Clinical study protocol. A multicenter, randomized, double-blind phase 2 trial of lenvatinib (E7080) in subjects with 131I-refractory differentiated thyroid cancer to evaluate whether an oral starting dose of 20 mg or 14 mg daily will provide comparable efficacy to a 24-mg starting dose, but have a better safety profile. 2015 Mar 20.

38 Aschebrook-Kilfoy B, Ward MH, Della Valle CT, Friesen MC. Occupation and thyroid cancer. Occup Environ Med. 2014;71(5):366-80.

39 Lope V, Perez-Gomez B, Aragones N, Lopez-Abente G, Gustavsson P, Plato N, et al. Occupational exposure to chemicals and risk of thyroid cancer in Sweden. Int Arch Occup Environ Health. 2009;82(2):267-74.

40 Taruscio D, Vittozzi L, Stefanov R. National plans and strategies on rare diseases in Europe. Adv Exp Med Biol. 2010;686:475-91.

41 Italian National Institute of Health. EUROPLAN project: EUROPLAN 2012-2015. On EUROPLAN website. 2015. <http://www.europlanproject.eu/Content?folder=1&content=1>. Accessed 2015 Mar 24.

42 Taruscio D, Agresta L, Amato A, Bernardo G, Bernardo L, Braguti F, et al. The Italian National Centre for Rare Diseases: where research and public health translate into action. Blood Transfus. 2014;12 Suppl 3:s591-s605.

43 Centro de Investigation Biomedica en Red de Enfermedades Raras. On Centro de Investigation Biomedica en Red de Enfermedades Raras (CIBERER) website. 2015. <http://www.ciberer.es/>.

44 Réseau CHU. L'information des CHU et de la conference des directeurs generaux de CHRU. On Haute Autorité de Santé (HAS) website. 2015. <http://www.reseau-chu.org/fileadmin/reseau-chu/anciens-articles/_new/maladies_rares.htm>.

45 Rodwell C, Aymé S. 2014 Report on the state of the art of rare disease activities in Europe: Part V - Activities in EU Member States and other European countries in the field of rare diseases. On European Union Committee of Experts on Rare Diseases (EUCERD) website. 2014. <http://www.eucerd.eu/upload/file/Reports/2014ReportStateofArtRDActivitiesV.pdf>. Accessed 2015 Apr 1.

46 Brose MS, Smit J, Capdevila J, Elisei R, Nutting C, Pitoia F, et al. Regional approaches to the management of patients with advanced, radioactive iodine-refractory differentiated thyroid carcinoma. Expert Rev Anticancer Ther. 2012;12(9):1137-47.

47 Robbins RJ, Wan Q, Grewal RK, Reibke R, Gonen M, Strauss HW, et al. Real-time prognosis for metastatic thyroid carcinoma based on 2-[18F]fluoro-2-deoxy-D-glucose-positron emission tomography scanning. J Clin Endocrinol Metab. 2006;91(2):498-505.

48 Carhill AA, Cabanillas ME, Jimenez C, Waguespack SG, Habra MA, Hu M, et al. The noninvestigational use of tyrosine kinase inhibitors in thyroid cancer: establishing a standard for patient safety and monitoring. J Clin Endocrinol Metab. 2013;98(1):31-42.

49 Eisai Inc. Prescribing information. Lenvima. 2015 Feb.

50 Busaidy NL, Cabanillas ME. Differentiated thyroid cancer: management of patients with radioiodine nonresponsive disease. J Thyroid Res. 2012;2012:618985.

51 McCabe C, Edlin R, Round J. Economic considerations in the provision of treatments for rare diseases. Adv Exp Med Biol. 2010;686:211-22.

52 Harney M, Richetta P, Atella V, et al. Acting together: A roadmap for sustainable healthcare. On European Steering Group onSustainable Healthcare (ESG) website. 2015. <http://static.diariomedico.com/docs/2015/03/20/ue-libro-blanco.pdf>. Accessed 2015 Mar 24.

53 European Medicines Agency. Guideline on the evaluation of anticancer medicinal products in man. On European Medicines Agency website. 2010. <http://www.ema.europa.eu/docs/en_GB/document_library/Scientific_guideline/2011/12/WC500119966.pdf>. Accessed 2012 Aug 23.

54 U.S.Food and Drug Administration. Guidance for industry clinical trial endpoints for the approval of cancer drugs and biologics. On U.S.Food and Drug Administration website. 2007. <http://www.fda.gov/downloads/Drugs/GuidanceComplianceRegulatoryInformation/Guidances/ucm071590.pdf>. Accessed 2011 Sep 27.

55 Institut de Veille Sanitaire. Évolution de l'incidence du cancer de la thyroïde en France métropolitaine - Bilan sur 25 ans. On Institut de veille sanitaire website. 2011. <http://www.invs.sante.fr/publications/2011/bilan_cancer_thyroide/>. Accessed 2015 Mar 26.

56 Colonna M, Mitton N, Grosclaude P. Estimation de la prévalence (partielle et totale) du cancer en France métropolitaine chez les 15 ans et plus en 2008 - Étude à partir des registres des cancers du réseau Francim. On Institut National du Cancer website. 2014. <http://www.e-cancer.fr/Expertises-et-publications/Catalogue-des-publications/Estimation-de-la-prevalence-partielle-et-totale-du-cancer-en-France-metropolitaine-chez-les-15-ans-et-plus-en-2008>.

57 Ceresini G, Corcione L, Michiara M, Sgargi P, Teresi G, Gilli A, et al. Thyroid cancer incidence by histological type and related variants in a mildly iodine-deficient area of Northern Italy, 1998 to 2009. Cancer. 2012;118(22):5473-80.

## Analysis of the Quality of Evidence

A critical analysis of the quality of each study is performed using the instruments below. These analyses involve commenting on all dimensions of the tool and providing a critical overview of the study and a quality grade.

**Overall assessment of the clinical program**

| **Type of evidence** | **Questions** | **Critical overview** | **Quality grade** |
| --- | --- | --- | --- |
| **Overall assessment of efficacy / effectiveness, safety and patient-reported outcomes data** | How relevant is the research program with regard to interventional and observational data? Are conclusions valid over the range of studies (conclusions across studies consistent or conflicting)?  Are individual trials relevant and valid? COMPLETE INSTRUMENT ABOVE FOR EACH INDIVIDUAL STUDY | The clinical program of lenvatinib for the treatment of progressive RR-DTC is limited to a single-arm phase 2 trial (excluded from this assessment) and a phase 3 randomized placebo-controlled trial (SELECT trial; the subject of this assessment). Supportive information is provided by an indirect treatment comparison versus sorafenib (the only tyrosine kinase inhibitor approved for progressive RR-DTC).  The SELECT trial is well designed and relevant in terms of target population, size, time horizon, and efficacy/safety outcomes selection. Patient-relevant outcomes (patient-reported outcomes, quality of life), however, were not assessed in this trial. Results of secondary endpoints (overall survival [78% at 24 months] and response rate [50%]) are consistent with the phase 2 trial results.[37] | 1  Low  2  Moderate  3  High  4  Excellent |

**Interventional studies**

**Study:** SELECT; phase 3, randomized, double-blind, placebo controlled (Schlumberger et al., 2015)[10]

**Disease:** progressive, locally advanced or metastatic

**Interventions:** lenvatinib 24 mg/day versus placebo

**Setting:** multicentre clinical trial

|  | | **Questions** | **Critical overview** | | **Quality grade** |
| --- | --- | --- | --- | --- | --- |
| **Overall assessment of efficacy / effectiveness, safety and patient-reported outcomes data** | | Is the study question relevant (choice of comparator, time horizon, patient population, and outcome measures)?  Is the design appropriate with a low risk of bias (setting & design, sample size, patient allocation, analyses, statistics)?  *See dimensions below* | This study was appropriately designed to explore the efficacy of an oncology drug, with standard outcomes measured. Reporting was clear with very few gaps (assessment method of relationship between AEs and intervention not provided).  Comparator was placebo although an active comparator (sorafenib) is approved for the same indication. In the absence of a head-to-head trial comparison, an indirect comparison of lenvatinib versus sorafenib was performed based on clinical data from the SELECT trial (not reported in the trial publication). | | 1  Low  2  Moderate  3  High  4  Excellent |
|  | **Dimension** | **Questions** | | **Comment** | |
| **1** | **Target population** | Are population characteristics fully reported? Is the setting clearly described?  Is the target population relevant (age, gender, disease stage, comorbidities, inclusion criteria/exclusion criteria, setting etc.)? Does it correspond to the actual population in which the intervention is envisioned to be used? | | Population characteristics are reported.  Study population corresponds to actual population targeted by the indication: progressive, locally advanced or metastatic RR-DTC. Patient characteristics are relevant. | |
| **2** | **Intervention & comparators** | Is the intervention in agreement with expected use? Does the choice of comparators reflect standard of care? | | Intervention is used as per indication.[32,49] Comparator is placebo, which corresponds to best supportive care (TSH suppression and palliative treatments; recommended by clinical practice guidelines[25]).  An active comparator would be sorafenib (same class drug indicated for RR-DTC; recommended by clinical guidelines[14,22,23,25]); in the absence of a head-to-head trial, an indirect treatment comparison of lenvatinib versus sorafenib was conducted based on clinical data from the SELECT trial (not reported in this publication). | |
| **3** | **Outcome measures** | Are all outcomes measures well defined (efficacy, safety and PRO)? Are the selected outcome measures (efficacy, safety and PRO) relevant? Are rationales for outcomes selection reported? Are they valid?  Are the instruments/methods/units used to measure outcomes reported? Are the instruments/methods/units used to measure outcomes (efficacy, PROs) valid? | | Efficacy and safety outcomes are well defined. PRO outcomes were not collected in this trial.  Selected primary and secondary efficacy endpoints (i.e. progression-free survival, overall survival, response rate) are valid and relevant outcomes, recommended by regulatory agencies (FDA and CHMP[53,54]) for oncology trials.  Rationale for outcomes selection was not given.  Assessment methods and units are reported. | |
| **4** | **Study design** | Was the sample size appropriate? Is the design appropriate? For RCTs, this includes: randomization; patient allocation concealment; blinding as appropriate; completeness of follow up; avoidance of carry-over effects. Were the groups similar at the outset of the study in terms of prognostic factors (e.g., severity of disease)? | | Sample size is appropriate (N=392; 261 in lenvatinib arm vs 131 placebo).  Study design (double-blind randomized controlled trial) is appropriate and well described.  Baseline characteristics were similar between the two treatment arms. | |
| **5** | **Adverse events** | Are methods for monitoring the occurrence of adverse events and assumptions with regard to which adverse events are related to the intervention(s) relevant and valid in light of previous experience with similar interventions/populations?  Are all relevant adverse events reported (deaths, serious, common, discontinuations), both treatment-related and all-cause? | | Methods for adverse event assessment are reported. However, details on how the relationship between AEs and the intervention was assessed are not provided.  All relevant adverse events are reported. | |
| **6** | **Time horizon** | Is time horizon long enough to capture all meaningful differences in key outcomes between the intervention and comparators? (e.g., Was the study stopped prematurely because of observed benefits or harms?) | | Time horizon was appropriate: follow-up of 17.1 months for 1^st^ data cut-off and approx. 34 months for 2^nd^ dataset – sufficient to capture effect of treatment on survival of cancer patients. | |
| **7** | **Analyses** | Are the analyses clearly described?  Are the analyses appropriate and comprehensive with sound and relevant statistics (e.g., intention-to-treat; missing data; control of confounding; subgroup analyses)? | | Analyses are clearly described in methodology section.  Analyses are comprehensive and statistics relevant: intent-to-treat and per protocol populations; subgroup analyses for primary outcome; sound statistics (Kaplan-Meier and log-rank test…); adjustment for cross-over effect conducted (rank-preserving structural failure time method). | |
| **8** | **Results (precision and strength of effect)** | Are results clearly reported, including patient disposition (population flow)?  Are differences observed meaningful (clinically and statistically)? Is there evidence of a dose-response relationship? | | Results including patient disposition (flow diagram) are clearly reported.  Statistical significance was reached on most outcomes. Clinical meaningfulness of results was not explicitly discussed.  Dose-response was not explored in this trial. | |
| **9** | **Conclusion** | Are the conclusions supported by the results? | | Study conclusions on the efficacy and safety profile of lenvatinib are supported by the results. | |

## Evidence tables

**Table 1: Pivotal safety and efficacy trials – design and efficacy results**

| Study | Design | Population | Interventions | Outcomes | Findings |
| --- | --- | --- | --- | --- | --- |
| **SELECT**  **Schlumberger et al., 2015[10]** | Phase 3  Multicenter, randomized, double-blind, placebo controlled  Analysis: ITT; per-protocol  Treatment duration, median months: 13.8 (lenvatinib); 3.9 (placebo)  Run-in period: NA  Follow-up: 17.1 months (median) | N= 392 (261 lenvatinib/ 131 placebo)  Inclusion criteria:   - Adults with histologically or cytologically confirmed diagnosis of one of the DTC subtypes of papillary thyroid cancer or follicular thyroid cancer - Measurable disease (central radiographic review) - Disease progression within prior 13 months (RECIST criteria) - RR-DTC defined by ≥1 measurable lesion with no iodine uptake ; and/or ≥1 measurable lesion that progressed within 12 months of radioiodine therapy, despite demonstration of radioiodine avidity at time of treatment (subjects not eligible for possible curative surgery); and/or cumulative activity of radioiodine of > 600 mCi or 22 GBq, with last dose administered ≥ 6 months prior to study entry - ≤1 prior VEGF/VEGFR-targeted therapy - Clinically stable brain metastases - Thyroxine suppression therapy and TSH not elevated - < Grade 2 severity existing chemotherapy- or radiation-related toxicities, except for alopecia and infertility - ECOG 0 to 2 - Adequately controlled blood pressure - Adequate renal, liver, bone marrow and blood coagulation function   Exclusion criteria:   - Anaplastic or medullary thyroid carcinoma - ≥ 2 prior VEGF/ VEGFR-targeted therapies - RR-DTC treatment other than TSH-suppressive thyroid hormone therapy   Key baseline characteristics   - Age, median yrs: 64 (lenvatinib); 61 (placebo) - Male: 48% (lenvatinib); 57% (placebo) - Diagnosis: papillary thyroid cancer (54.0% lenvatinib, 54.2% placebo); follicular (35.2% lenvatinib, 31.3% placebo); poorly differentiated cancer (10.7% lenvatinib, 14.5% placebo) - ECOG 0-1: 96% - Metastases: 89% pulmonary, 39% bone metastases.   Attrition:   - Completed study drug: | Product: lenvatinib 24 mg/day  Comparator: placebo  28-day cycle | Primary endpoint:  Progression-free survival (PFS; time from randomization to the first documentation of disease progression by independent radiologic review or to death in the ITT population)  Secondary endpoints:   - Overall survival (OS; time from to death from any cause) - Objective response rate (complete + partial response; RECIST) - Safety and tolerability - Pharmacokinetic profile   *Exploratory*   - Disease control rate (DCR; complete or partial response or stable disease) - Clinical benefit rate (CBR; complete or partial response or durable stable disease for > 23 weeks) | \|  \| **Lenvatinib**  **N = 261** \| **Placebo**  **N = 131** \| \| --- \| --- \| --- \| \| **Primary: Progression-free survival** \| \| \| \| Primary analysis, IRR and ITT populations* \|  \|  \| \| Median (95% CI), mo \| 18.3  (15.1–NE) \| 3.6  (2.2–3.7) \| \| HR (95% CI) \| 0.21 (0.14–0.31); *P*< .001 \| \| \| Rate, % (95% CI) \|  \|  \| \| 6 mo \| 77.5  (71.7–82.3) \| 25.4  (18.0–33.6) \| \| 12 mo \| 63.0  (56.5–68.9) \| 10.5  (5.7–16.9) \| \| 18 mo \| 51.1  (43.3–58.3) \| 3.8  (1.1–9.2) \| \| 24 mo \| 44.3  (35.1–53.1) \| NE \| \| Prespecified sensitivity analyses \|  \|  \| \| Investigator assessment, ITT \|  \|  \| \| Median (95% CI), mo \| 16.6  (14.8–NE) \| 3.7  (3.5–5.4) \| \| HR (95% CI) \| 0.24 (0.16–0.35) ; *P*< .001 \| \| \| IRR population \|  \|  \| \| Median (95% CI), mo \| 16.6  (14.8–20.3) \| 3.6  (2.2–3.7) \| \| HR (95% CI) \| 0.22 (0.15–0.32) ; *P*< .001 \| \| \| ***Secondary endpoints*** \| \| \| \| **Overall survival**, RPSFT adjusted, ITT \|  \|  \| \| *First data cut-off* \|  \|  \| \| Median (95% CI), mo \| NE  (22.0–NE) \| NE  (14.3–NE) \| \| HR (95% CI) \| 0.62 (0.40–1.00); *P=*.05 \| \| \| Rate, RPSFT adjusted, % (95% CI) \|  \|  \| \| 6 mo \| 90.7  (86.4–93.7) \| 85.3  (78.0–90.4) \| \| 12 mo \| 81.6  (76.2–85.8) \| 70.0  (57.1–79.7) \| \| 18 mo \| 72.3  (65.7–77.9) \| 63.0  (44.3–76.9) \| \| 24 mo \| 58.2  (46.0–68.6) \| NE \| \| *Second data cut-off[31]* \|  \|  \| \| Median (95% CI), mo \| NE  (30.9–NE) \| 19.1  (14.3–NE) \| \| HR (95% CI) \| 0.53 (0.34–0.82); *P*< .0051 \| \| \| **Response rate, n (%)** \| 169 (64.8) \| 2 (1.5) \| \| Odds ratio (95% CI) \| 28.87 (12.46–66.86); *P*< .001 \| \| \| Complete response \| 4 (1.5) \| 0 \| \| Partial response \| 165 (63.2) \| 2 (1.5) \| \| Stable disease \| 60 (23.0) \| 71 (54.2) \| \| Durable stable disease ≥23 wk \| 40 (15.3) \| 39 (29.8) \| \| Progressive disease \| 18 (6.9) \| 52 (39.7) \| \| Could not be evaluated \| 14 (5.4) \| 6 (4.6) \| \| ***Exploratory endpoints*** \| \| \| \| **Disease-control rate, n (%)** \| 229 (87.7) \| 73 (55.7) \| \| Odds ratio (95% CI) \| 5.05 (2.98–8.54)§ \| \| \| **Clinical-benefit rate, n (%)** \| 209 (80.1) \| 41 (31.3) \| \| Odds ratio (95% CI) \| 7.63 (4.55–12.79); *P*< .001 \| \| \| **Time to first objective response** \|  \|  \| \| Median (95% CI) , mo \| 2.0  (1.9–3.5) \| 5.6  (1.8–9.4) \|   *identical results with per-protocol analyses |
| CI: confidence interval; HR: hazard ratio; IRR: independent radiologic review; ITT: intention-to-treat; NE: not estimable; RPSFT: rank-preserving structural failure time; RR-DTC: Radioiodine-refractory/resistant differentiated thyroid cancer. | | | | | |

**Criteria for tumor response in SELECT trial**

| **Response Criteria** | **Evaluation of Lesions** |
| --- | --- |
| *Evaluation of target lesions* |  |
| Complete Response (CR) | Disappearance of all target lesions. Any pathological lymph nodes (whether target or non‐target) must have reduction in short axis to < 10 mm. |
| Partial Response (PR) | At least a 30% decrease in the sum of diameters of target lesions, taking as reference the baseline sum of diameters. |
| Stable Disease (SD) | Neither sufficient shrinkage to qualify for PR nor sufficient increase to qualify for PD, taking as reference the smallest sum of diameters while on study. |
| Progressive Disease (PD) | At least a 20% increase in the sum of diameters of target lesions, taking as reference the smallest sum on study (this includes the baseline sum if that is the smallest on study). In addition to the relative increase of 20%, the sum must also demonstrate an absolute increase of at least 5mm. |
| Not Evaluable (NE) | Progression has not been documented and one or more target lesions have not been assessed or have been assessed using a different method than baseline that makes comparability impossible. |
| *Evaluation of non-target lesions* |  |
| Complete Response (CR) | Disappearance of all non‐target lesions. All lymph nodes must be non‐pathological in size (< 10 mm short axis). |
| Non-complete/non-partial disease | Persistence of one or more non‐target lesion(s). |
| Progressive Disease (PD) | Unequivocal progression of existing non‐target lesions. |
| Not Evaluable (NE) | Progression has not been documented and one or more non‐target lesions have not been assessed or have been assessed using a different method than baseline that makes comparability impossible. |
| **Source**: Schlumberger et al, 2015.[10] | |

## Additional data – EPIDEMIOLOGY

| Data Source /  *Country (region)* | Data |
| --- | --- |
| Institut de Veille Sanitaire[55]  *France* | Annual incidence:  Papillary cancer: females 10.6/100,000; males 3.0/100,000 (2002–2008)  Follicular cancer: females 1.2/100,000; males 0.5/100,000 (2002–2008) |
| FRANCIM network of cancer registries[56]  *France* | Prevalence of thyroid cancer, persons aged ≥ 15 years:   - Women: 69,559; men: 19,236 (2008) |
| Tumour Registry of Parma[57]  *Italy (Parma province)* | Annual incidence:  Papillary cancer: 9.03/100,000 (2004–2009)  Follicular cancer: 1.26/100,000 (2004–2009)  Hürthle cell cancer: 0.84/100,000 (2004–2009) |
| GLOBOCAN[20]  *Italy* | Thyroid cancer 5-year prevalence:   - Adults: 39,082 cases (2012) |
| GLOBOCAN[19]  *Spain* | Thyroid cancer 5-year prevalence:   - Adults: 8,097 (2012) |
