# Supplementary material for: Appraising the holistic value of Lenvatinib for radio-iodine refractory differentiated thyroid cancer: A multi-country study applying pragmatic MCDA
Source: BMC Cancer. 2017 Apr 17;17:272. doi: 10.1186/s12885-017-3258-9 (PMC5393009; doi:10.1186/s12885-017-3258-9)
Supplement: Supplementary file 4 — Recruitment criteria for panelists. (DOCX 31 kb) [file 12885_2017_3258_MOESM4_ESM.docx]

# Additional File 4: Recruitment criteria for panelists and panel compositions

**RECRUITMENT CRITERIA**

| Type of panelist | Criteria for Recruitment |
| --- | --- |
| Patient representative | - Active member of a local patient support group in the relevant disease area (ideally thyroid cancer, otherwise solid organ cancer in general) |
| Specialist clinician | - Active practitioner (oncologist) specializing in treating patients with differentiated thyroid cancer (DTC), including radioiodine-refractory DTC - Working at a renowned DTC treatment center - Author of prominent guidelines or other publications in the field - No conflict of interest |
| Health economists/Epidemiologists | - Academic/consulting with active research program relevant to the field of decisionmaking |
| Policy decisionmakers | - Previous member of a relevant HTA decision- or recommendation-making body (Note: current members will not be able to participate because of conflict of interest) - Ideally involved in an active research program in decisionmaking |

**PANEL COMPOSITIONS**

| Type of panelist | **France** | **Italy** | **Spain** |
| --- | --- | --- | --- |
| Patient representative | 1 | 1 | 1 |
| Specialist clinician | 4 | 2 | 2 |
| Health economists/Epidemiologists | 2 | 2 | 1 |
| Policy decisionmakers | 1 | 3 | 4 |
