# Supplementary material for: Appraising the holistic value of Lenvatinib for radio-iodine refractory differentiated thyroid cancer: A multi-country study applying pragmatic MCDA
Source: BMC Cancer. 2017 Apr 17;17:272. doi: 10.1186/s12885-017-3258-9 (PMC5393009; doi:10.1186/s12885-017-3258-9)
Supplement: Supplementary file 5 — Exploratory analysis of weights by category of panelists. (DOCX 33 kb) [file 12885_2017_3258_MOESM5_ESM.docx]

# Additional File 5: Exploratory analysis of weights by category of panelists

**Ranking of decision criteria by mean normalized weight (SD**)

| **Rank** | **Policy decisionmaker  (n=8)** | | **Specialist clinician  (n=8)** | | **Health Economist / Epidemiologist (n=5)** | | **Patient Representative  (n=3)** | |
| --- | --- | --- | --- | --- | --- | --- | --- | --- |
| 1 | Comparative effectiveness ­ | 0.114 (0.017) | **Disease severity** | 0.103 (0.013) | Comparative effectiveness ­ | 0.111 (0.011) | **Disease severity** | 0.101 (0.021) |
| 2 | *Type of therapeutic benefit* | 0.096 (0.022) | Quality of evidence | 0.098 (0.015) | Size of affected population | 0.097 (0.020) | Comparative safety/tolerability | 0.093 (0.008) |
| 3 | Quality of evidence | 0.096 (0.021) | Comparative effectiveness ­ | 0.098 (0.012) | **Disease severity** | 0.094 (0.033) | Unmet needs | 0.092 (0.016) |
| 4 | **Disease severity** | 0.090 (0.017) | Unmet needs | 0.095 (0.027) | Unmet needs | 0.093 (0.024) | Comparative patient-perceived health/PROs | 0.092 (0.016)* |
| 5 | Comparative safety/tolerability | 0.087 (0.018) | *Type of therapeutic benefit* | 0.091 (0.017) | Comparative cost consequences - cost of intervention | 0.093 (0.015) | *Type of therapeutic benefit* | 0.092 (0.016)* |
| 6 | Type of preventive benefit | 0.081 (0.024) | Type of preventive benefit | 0.086 (0.021) | Comparative safety/tolerability | 0.093 (0.020) | Comparative effectiveness ­ | 0.087 (0.12)† |
| 7 | Comparative patient-perceived health/PROs | 0.080 (0.026) | Comparative safety/tolerability | 0.085 (0.019) | Quality of evidence | 0.085 (0.034) | Quality of evidence | 0.087 (0.012)† |
| 8 | Comparative cost consequences - cost of intervention | 0.078 (0.021) | Comparative cost consequences - cost of intervention | 0.079 (0.021) | Type of therapeutic benefit | 0.083 (0.011) | Size of affected population | 0.080 (0.018) |
| 9 | Size of affected population | 0.075 (0.028) | Comparative patient-perceived health/PROs | 0.072 (0.018) | Comparative patient-perceived health/PROs | 0.075 (0.018) | Expert consensus/CPGs | 0.079 (0.025) |
| 10 | Unmet needs | 0.072 (0.015) | Expert consensus/CPGs | 0.067 (0.025) | Expert consensus/CPGs | 0.066 (0.013) | Type of preventive benefit | 0.079 (0.006) |
| 11 | Expert consensus/CPGs | 0.072 (0.027) | Comparative cost consequences - other costs | 0.065 (0.016) | Type of preventive benefit | 0.064 (0.030) | Comparative cost consequences - other costs | 0.062 (0.028) |
| 12 | Comparative cost consequences - other costs | 0.060 (0.028) | Size of affected population | 0.062 (0.020) | Comparative cost consequences - other costs | 0.047 (0.25) | Comparative cost consequences - cost of intervention | 0.056 (0.017) |

* Criteria tied for third rank; † criteria tied for fourth rank. **Bold** text indicates criterion ranked among the five-highest weighted, by each of the four categories of stakeholder. *Italics* indicates criterion ranked among the five-highest weighted, by three of the four categories of stakeholder.

SD: standard deviation
